# The Role of Triterpenoids in Gastric Ulcer: Mechanisms and Therapeutic Potentials

**DOI:** 10.3390/ijms26073237

**Published:** 2025-03-31

**Authors:** Congcong Shen, Shengyu Zhang, Han Di, Shuang Wang, Yanhong Wang, Feng Guan

**Affiliations:** 1 School of Pharmacy, Heilongjiang University of Chinese Medicine, Harbin 150040, China; scc16637647314@163.com (C.S.); tt13892638691@163.com (S.Z.); 15846488827@163.com (H.D.); swhljucm@163.com (S.W.); 2 Key Laboratory of Basic and Application Research of Beiyao, Ministry of Education, Heilongjiang University of Chinese Medicine, Harbin 150040, China

**Keywords:** triterpenoids, gastric ulcer, pharmacological mechanism, gastroprotective

## Abstract

Gastric ulcer (GU) is a prevalent gastrointestinal disorder impacting millions worldwide, with complex pathogenic mechanisms that may progress to severe illnesses. Conventional therapies relying on anti-secretory agents and antibiotics are constrained by drug abuse and increased bacterial resistance, highlighting the urgent need for safer therapeutic alternatives. Natural medicinal compounds, particularly triterpenoids derived from plants and herbs, have gained significant attention in recent years due to their favorable efficacy and reduced toxicity profiles. Emerging evidence indicates that triterpenoids exhibit potent anti-ulcer properties across various experimental models, modulating key pathways involved in inflammation, oxidative stress, apoptosis, and mucosal protection. Integrating current knowledge of these bioactive compounds facilitates the development of natural triterpenoids, addresses challenges in their clinical translation, deepens mechanistic understanding of GU pathogenesis, and drives innovation of therapeutic strategies for GU. This review comprehensively evaluates the progress of research on triterpenoids in GU treatment since 2000, discussing their biological sources, structural characteristics, pharmacological activities, and mechanisms of action, the animal models employed in the studies, current limitations, and the challenges associated with their clinical application.

## 1. Introduction

**Figure 1:**
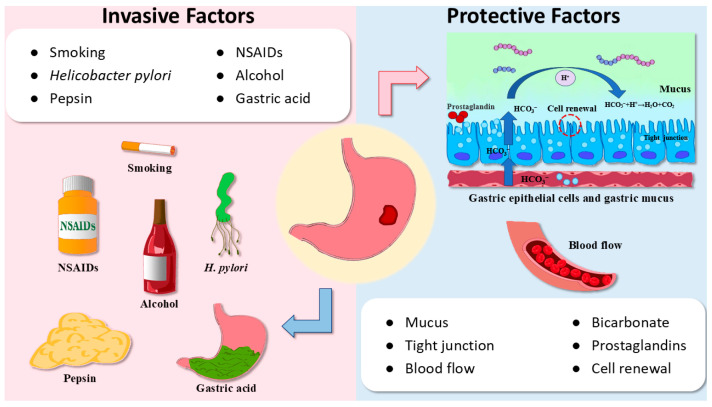
Invasive factors and protective factors affecting the incidence of GU. Protective factors include mucus, bicarbonate, tight junction, prostaglandins, blood flow, and cell renewal. Invasive factors include smoking, NSAIDs, *H. pylori*, alcohol, pepsin, and gastric acid.

Gastric ulcer (GU) is an injury of mucosal integrity resulting from diverse invasive factors traversing the muscularis mucosae layer [1]. This widespread condition impacts nearly 10% of individuals globally, with potential progression to severe bleeding, perforation, and obstruction that collectively threaten patient well-being [2]. The causes of the emergence and development of GU are diverse. Recent studies highlight the disrupted equilibrium between invasive factors (e.g., smoking habit, alcohol consumption, pepsin, and gastric acid) and protective factors (e.g., mucus, bicarbonate, tight junction, and prostaglandins) as the pivotal determinant underlying ulcer progression [3,4,5,6]. In the past, GU was commonly attributed to excessive gastric acid secretion, stress, and poor dietary habits. Nowadays, *Helicobacter pylori* (*H. pylori*) infection and the extensive use of non-steroidal anti-inflammatory drugs (NSAIDs) have become the main triggers of GU [7] (Figure 1). The presence of GU suggests that there is inflammation in the gastric mucosa, and the severity of inflammation is related to the proximity of the ulcer center. A persistent inflammatory environment will eventually lead to gastric cancer [8,9].

Treatment of GU is usually aimed at healing the ulcer, preventing the development of complications, and inhibiting recurrence of the ulcer. Current treatment of GU is based on the use of anti-secretory agents and antibiotics that eradicate *H. pylori*. Anti-secretory agents encompass antiacids, histamine H2 receptor antagonists, and proton pump inhibitors. Proton pump inhibitors are the most rapid and potent, establishing them as first-line therapeutic agents for GU [10,11]. However, with the extensive clinical use of these drugs, several problems have ensued. Proton pump inhibitors demonstrate excessive utilization in clinical settings, with persistent post-discharge administration for nonessential therapeutic indications correlating with elevated risks of acute kidney injury, chronic kidney disease, and fractures [12]. Furthermore, the extensive utilization of antibiotics not only enhances *H. pylori* resistance but also increases the prevalence of diseases that are negatively associated with *H. pylori*, such as gastroesophageal reflux and obesity [13]. The search for anti-ulcer drug candidates from natural medicines represents an effective approach to addressing GU.

Natural medicines have demonstrated their safety and efficacy in both traditional and modern medicine, making them an ideal source of compounds with anti-ulcer potential. In recent years, bioactive compounds derived from natural sources, such as polysaccharides [14], flavonoids [15], coumarin [16], and alkaloids [17], have demonstrated significant anti-ulcer activities. In addition to these compounds, natural terpenoids have garnered considerable attention in the treatment of GU due to their unique structural characteristics and diverse biological activities.

Terpenoids originate through the mevalonate pathway biosynthesis and are characterized by isoprene units, which constitute the core backbone architecture of these specialized metabolites. Terpenoids demonstrate remarkable architectural diversity in molecular frameworks, underpinning their extensive bioactive potential. Particularly, triterpenoid derivatives exhibit the most therapeutically significant pharmacological profiles within all terpenoids. Triterpenoids are made up of six isoprene units with a skeleton containing 30 carbon atoms. Most of the natural triterpenoids are ring-forming, with tetracyclic and pentacyclic derivatives predominating. Depending on the number of rings and the manner of ring condensation, triterpenoids can be categorized into dammarane-type, cycloartane-type, tirucallane-type, oleanane-type, ursane-type, lupane-type, and friedelane-type. Oxidation, methylation, and acylation at varying positions on the skeleton further enrich the structure of triterpenoids, resulting in a wide range of pharmacological properties, such as anti-inflammatory, antioxidant, antimicrobial, anti-cancer, and cardioprotective effects [18,19,20,21,22].

In recent years, numerous triterpenoids isolated from natural medicines have been demonstrated to possess gastroprotective efficacy within a diverse array of GU models. However, this area remains critically under-reviewed. Our search identified only one paper in 1998 focusing on the synthesis and anti-ulcer activities of glycyrrhizin, oleanolic acid, and ursolic acid derivatives, specifically examining these particular derivatives rather than triterpenoids as a broader class [23]. With advancing research, scholars have made remarkable progress in exploring triterpenoids for GU treatment, yet no comprehensive review has analyzed or synthesized findings in this field over the past two decades. Therefore, this review consolidates research on triterpenoids in GU treatment since 2000, using studies retrieved from reference databases such as PubMed, Science Direct, Scopus, and Google Scholar. Specifically, it includes (1) the natural origins, chemical structures, and biological activities of gastroprotective triterpenoids; (2) proposed pharmacological mechanisms with graphical schematics; (3) a summary table that integrates information on compounds, sources, models, activities, and mechanisms; (4) evaluation of GU animal models employed in the reviewed studies; and (5) current limitations and future challenges in clinical implementation. This review aims to provide a reference for the development and utilization of triterpenoids in medicinal plants as well as the improvement and updating of therapeutic regimens for GU.

## 2. The Activity of Triterpenoids in GU

### 2.1. Dammarane-Type Triterpenoids

#### 2.1.1. Ginsenoside Rb1, Ginsenoside Rd, and Ginsenoside Rg3

**Figure 2:**
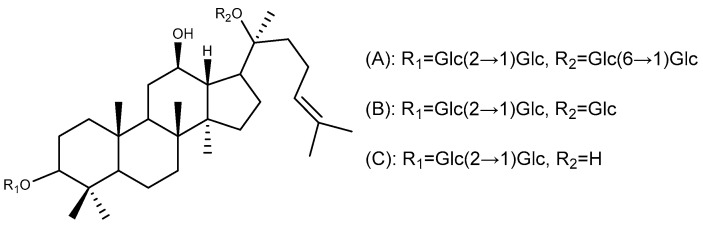
Protopanaxadiol type triterpenoids with anti-ulcer activity. (**A**) Ginsenoside Rb1; (**B**) Ginsenoside Rd; and (**C**) Ginsenoside Rg3.

Ginsenosides are the main active components of ginseng (*Panax ginseng* C. A. Meyer), San-Qi (*Panax notoginseng* (Burkill) F. H. Chen ex C. H. Chow), and American ginseng (*Panax quinquefolius* L.). They are structurally categorized into four chemotypes based on sapogenin skeletal frameworks: protopanaxadiol (ginsenosides Rb1, Rd, and Rg3) (Figure 2), protopanaxatriol (ginsenoside Rh4), ocotillol (pseudoginsenoside F11), and oleanane-type derivatives exemplified by Ro [24]. The first three of these types are all tetracyclic triterpenes of the dammarane type.

A substantial body of evidence from numerous studies indicates that ginsenoside Rb1, ginsenoside Rd, and ginsenoside Rg3 are capable of treating malignant tumors through multiple pathways [25]. Beyond that, the rich pharmacological properties they possess also make them exert a beneficial effect on the treatment of GU. In the hydrochloric acid/ethanol (HCl/EtOH)-induced GU model in rats, ginsenoside Rb1, isolated from the active fraction of ginseng head, exhibited a significant inhibitory effect on gastric mucosal damage by promoting gastric mucus secretion [26]. Ginsenoside Rd was isolated from ginseng flower buds through methanol extraction followed by successive normal-phase and reversed-phase silica gel column chromatography. In ethanol and indomethacin-induced gastric mucosal injury models, this compound exhibited ulcer inhibition rates of 57.6% and 52.1% (100 mg/kg, p.o.), respectively, and its efficacy was comparable to the reference agent cetraxate hydrochloride [27]. It has been shown that ginsenoside Rg3 (5, 10, and 20 mg/kg, p.o.) exhibited a dose-dependent reduction in the ulcer index in ethanol, pylorus ligation, and acetic acid-induced GU in rats. This effect was associated with a reduction in the levels of inducible nitric oxide synthase (iNOS) and endothelin-1 (ET-1), as well as an increase in the expression of superoxide dismutase (SOD), epidermal growth factor (EGF), and epidermal growth factor receptor (EGFR) [28].

#### 2.1.2. Ginsenoside Rh4 and Protopanaxatriol

**Figure 3:**
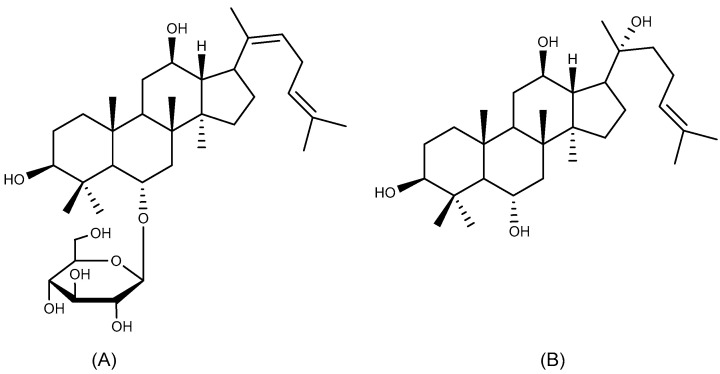
Protopanaxatriol type triterpenoids with anti-ulcer activity. (**A**) Ginsenoside Rh4 and (**B**) protopanaxatriol.

Ginsenoside Rh4 (Figure 3A) is a dammarane-type tetracyclic triterpenoid saponin that, in addition to its anti-tumor effects [29,30], has been shown to protect gastric tissues through a variety of pathways. Pretreatment with ginsenoside Rh4 (60 mg/kg) for a period of seven days was observed to significantly improve gastric mucosal histomorphology in ethanol-induced mucosal damage in rats. This therapeutic effect involves a multimodal mechanism: upregulating nitric oxide (NO) and prostaglandin E2 (PGE2) synthesis, concomitant with COX-2 expression downregulation and NF-κB transduction suppression, collectively attenuating mucosal oxidative damage and pro-inflammatory cascades. Additionally, the compound was shown to elevate B-cell lymphoma-2 (Bcl-2) levels and reduce Bcl-2-associated X protein (Bax) and Factor-related Apoptosis (Fas), which in turn inhibits apoptosis [31].

Protopanaxatriol (Figure 3B) is a sapogenin and a metabolite of ginsenoside Rh4, exhibiting significant anti-inflammatory and antioxidant activity [32,33]. The gastroprotective effects of protopanaxatriol were examined in an acetic acid-induced GU model. The pharmacodynamic evaluation revealed that medium and high doses of protopanaxatriol (10 and 20 mg/kg, p.o.) mitigated the loss of body weight and the expansion of ulcerated areas induced by acetic acid. Additionally, the levels of interleukin-6 (IL-6), malondialdehyde (MDA), tumor necrosis factor α (TNF-α), ET-1, EGF, and SOD were dose-dependently regulated. It is noteworthy that the modulatory effects of protopanaxatriol at a high dose were comparable to those of the positive control omeprazole [34].

#### 2.1.3. Ocotillol

**Figure 4:**
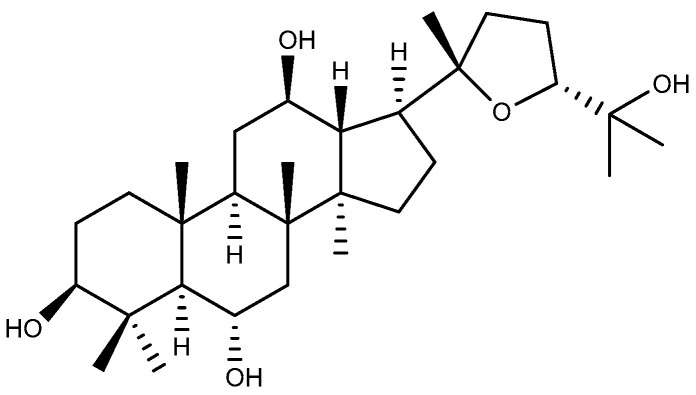
The structure of ocotillol.

Ocotillol-type saponins represent a distinctive class of dammarane-type saponins, characterized by the presence of a tetrahydrofuran ring. They are widely distributed in various ginseng species, including American ginseng and Vietnamese ginseng, serving as a key marker of American ginseng [35]. Ocotillol-type saponins demonstrate multifaceted therapeutic potential, such as anti-inflammatory, antibacterial, and anti-tumor effects [36]. Ocotillol (Figure 4) is the sapogenin of ocotillol-type saponins and their main metabolic component after oral administration. Ocotillol can reduce the level of ET-1 in serum and increase the level of NO in serum and gastric mucosa, as well as enhance the level of SOD, EGF, and EGFR in gastric mucosa. This ultimately leads to an improvement in the morphology of gastric tissues in model rats, which becomes similar to that of normal rats [37].

### 2.2. Cycloartane-Type Triterpenoid

**Figure 5:**
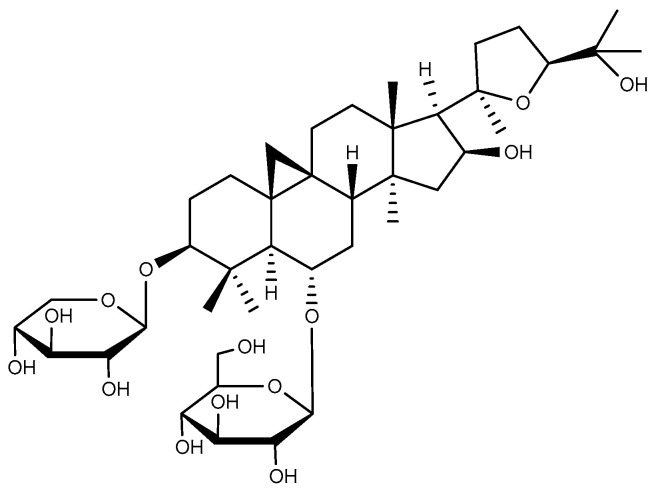
The structure of astragaloside IV.

*Astragalus membranaceus* (Fisch.) Bunge, a key medicinal plant in traditional Chinese medicine, has been widely utilized in clinical applications. Astragaloside IV (Figure 5) is a cycloartane-type tetracyclic triterpenoid saponin present in the aqueous extracts of *Astragalus membranaceus* and is the primary active component of the plant [38]. It exhibits a number of therapeutic profiles, mechanistically encompassing anti-inflammatory, antioxidant, anti-tumor, and neuroprotective effects [39]. In an ethanol-induced GU rat model, the protective effect of astragaloside IV was reversed by the NO synthase inhibitor L-NAME, indicating the involvement of NO in the anti-ulcer mechanism of astragaloside IV [40]. In addition, astragaloside IV (1, 10 and 50 mg/kg, p.o.) dose-dependently decreased gastric injury induced by water immersion and restraint stress in rats, with ulcer inhibition of 70.79% at a dose of 50 mg/kg. Besides enhancing the gastric mucosal barrier (increasing gastric pH and gastric mucus volume), anti-inflammatory (decreasing the levels of TNF-α and monocyte chemoattractant protein-1 (MCP-1)), and antioxidant (enhancing the activity of SOD and reducing the level of MDA) functions, astragaloside IV also upregulated the expression of heat shock protein 70 (HSP70) and thus inhibited the activation of Bax, showing an anti-apoptotic effect [41]. The administration of astragaloside IV (50 mg/kg, p.o.) inhibited an aspirin-induced decrease in cyclooxygenase-1 (COX-1) expression, thereby increasing the PGE2 level, enhanced the activity of SOD and the level of NO in the stomach, and attenuated the degree of aspirin-induced pathological damage to gastric mucosa. In vitro, aspirin-induced apoptosis was significantly inhibited by astragaloside IV (50 μg/mL). Notably, astragaloside IV did not interfere with aspirin’s effects on COX-2, suggesting its potential for concurrent administration with aspirin to mitigate the gastrointestinal side effects associated with long-term aspirin therapy [42].

### 2.3. Tirucallane-Type Triterpenoid

**Figure 6:**
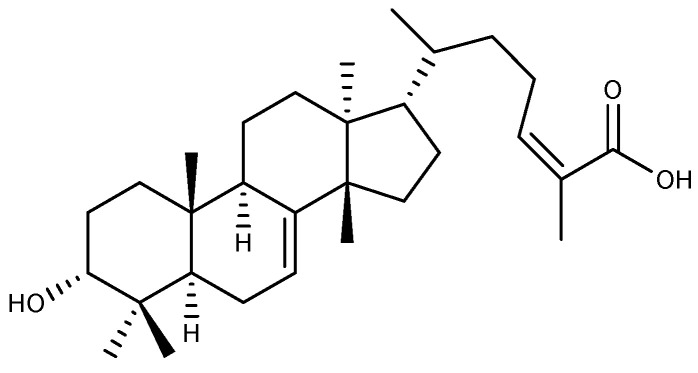
The structure of 3α-hydroxymasticadienoic acid.

*Amphipterygium adstringens* (Schltdl.) Schiede ex Standl is a medicinal plant native to Mexico that is considered an important anti-ulcer drug in local traditional medicine, and its stem bark is often used in treating various conditions like GU and gastritis [43]. 3α-Hydroxymasticadienoic acid (Figure 6), a tirucallane-type tetracyclic triterpenoid, was isolated from the dichloromethane fraction of the hexane extract of *Amphipterygium adstringens* stem bark via silica gel column chromatography. This compound exhibited a significant protective effect on gastric mucosa in the ethanol-induced GU model. This effect was inhibited by the sulfhydryl blocker N-ethylmaleimide, indicating that the anti-ulcer activity of 3α-hydroxymasticadienoic acid is dependent on endogenous sulfhydryl groups [44]. In addition, 3α-hydroxymasticadienoic acid has been shown to increase PGE2 levels and SOD activity while decreasing TNF-α and leukotriene B4 (LTB4) levels, exhibiting anti-inflammatory and antioxidant effects, as well as elevating the levels of gaseous mediators NO and hydrogen sulfide (H_2_S) in rat gastric tissue, which functions to safeguard the stomach mucosa against indomethacin injury [45].

### 2.4. Oleanane-Type Triterpenoids

#### 2.4.1. Oleanolic Acid

**Figure 7:**
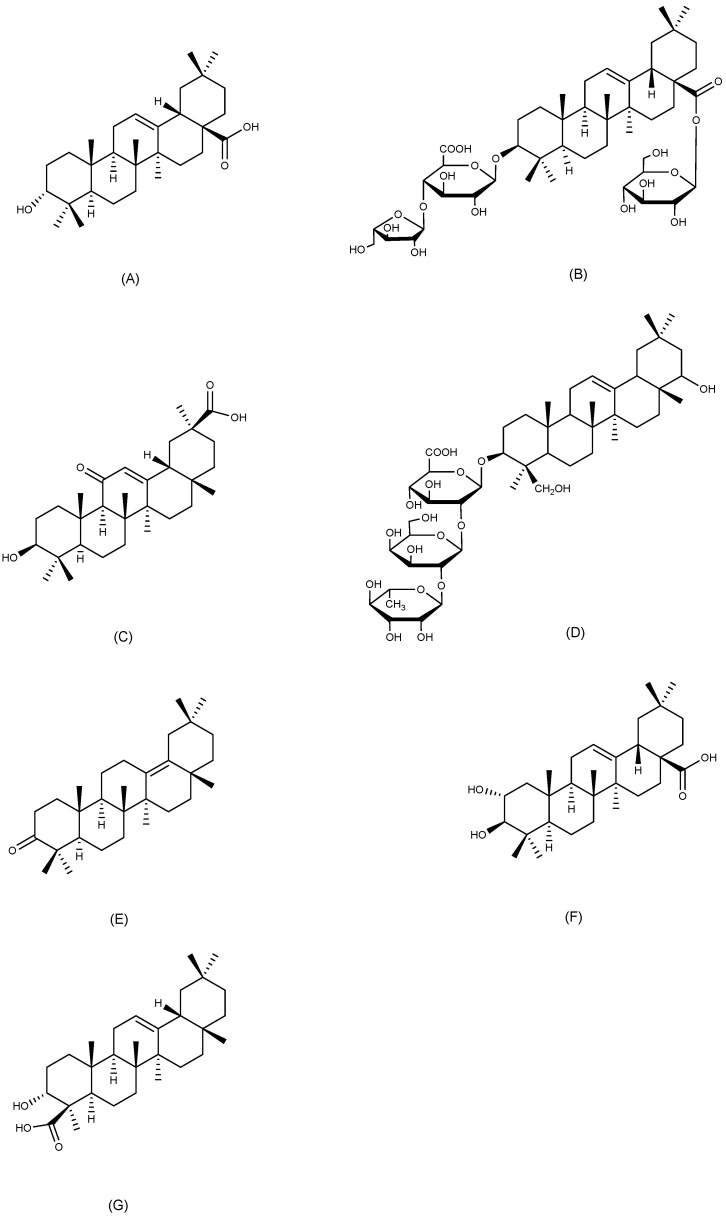
Oleanane-type triterpenoids with anti-ulcer activity. (**A**) Oleanolic acid; (**B**) Araloside A; (**C**) 18β-Glycyrrhetinic Acid; (**D**) Soyasaponin Bb; (**E**) δ-Amyrone; (**F**) Maslinic acid; and (**G**) α-Boswellic acid.

Oleanolic acid (Figure 7A) is an oleanane-type pentacyclic triterpenoid, widely present in nature and isolated from more than 1600 plant species, many of which serve as food and medicine. It is most prevalent in Oleaceae plants [46]. Oleanolic acid has attracted significant scientific attention because of its wide range of pharmacological effects, including gastroprotective, anti-inflammatory, antibacterial, antiviral, and anti-tumor properties [47]. The anti-ulcer effects of oleanolic acid (50, 100, and 200 mg/kg, p.o.) were shown in ethanol, aspirin, and pylorus ligation-induced GU models. In the last two models, its therapeutic effectiveness was similar to that of ranitidine at a dose of 50 mg/kg orally [48]. Specifically, it was observed that oleanolic acid (100 mg/kg, p.o.) markedly diminished the area of acetic acid-induced gastric lesions. In oleanolic acid-treated rats, the thickness of the gastric mucus layer increased to 540 μm, compared with 342 μm in control rats. These findings suggest that oleanolic acid exerts therapeutic effects on acetic acid-induced chronic GU in rats [49]. In vitro, oleanolic acid and its derivatives significantly reduced the damage caused by sodium taurocholate to human gastric adenocarcinoma cells (AGS), increased the content of PGE2 in AGS cell cultures, and substantially stimulated the proliferation of human lung fibroblasts. These gastroprotective activities of oleanolic acid and its derivatives were successfully validated in the HCl/EtOH-induced gastric injury model in mice. These activities attenuated gastric injury in mice to varying degrees [50].

#### 2.4.2. Araloside A

Araloside A (Figure 7B), an oleanane-type pentacyclic triterpenoid commonly present in Aralia plants, is nontoxic and has shown some antioxidant effects [51]. It is the primary anti-ulcer constituent of *Aralia elata* (Miq.) Seem. and demonstrated considerable inhibitory effects on HCl/EtOH, aspirin, pylorus ligation, and stress-induced GU in four distinct rat models. In the last model, araloside A (100 mg/kg, p.o.) showed a stronger anti-ulcer effect than cimetidine (100 mg/kg, p.o.), which was related to the inhibition of gastric acid secretion [52]. Similarly, in ethanol and aspirin-induced GU in mice, araloside A (40 mg/kg, p.o.) improved gastric blood flow, enhanced gastric mucosal defenses, and inhibited H^+^/K^+^-ATPase, thereby increasing gastric juice pH. It is noteworthy that araloside A exhibited a more potent anti-apoptotic effect compared to the positive control omeprazole by elevating the Bcl-2/Bax ratio, inhibiting cytochrome c release, and downregulating caspase-3 and caspase-9 activity as well as related mRNA expression [53].

#### 2.4.3. 18β-Glycyrrhetinic Acid

Licorice, which is derived from the dried roots and rhizomes of *Glycyrrhiza uralensis* Fisch., *Glycyrrhiza glabra* L., and *Glycyrrhiza inflata* Bat., has been utilized in traditional medicine worldwide for a long time. It is often used to treat GU and respiratory problems [54]. 18β-glycyrrhetinic acid (Figure 7C) is an oleanane-type pentacyclic triterpenoid, primarily derived from licorice, and it demonstrates various pharmacological activities, including anti-inflammatory, antioxidant, antimicrobial, and anti-tumor effects [55]. Previous research has shown that 18β-glycyrrhetinic acid exhibits significant anti-ulcer activity in a variety of GU models [56,57]. Furthermore, 18β-glycyrrhetinic acid showed inhibitory effects on *H. pylori* in vitro and in vivo. Among various licorice extracts, 18β-glycyrrhetinic acid demonstrated the most powerful inhibition of *H. pylori* strains derived from gastric biopsies of patients with GU, with the effect being both rapid and dose-dependent [58]. The administration of 18β-glycyrrhetinic acid significantly reduced ulcer scores, improved gastric histomorphometry, inhibited *H. pylori*-induced gastric juice pH elevation, and decreased the expression levels of COX-2, interleukin-1β (IL-1β), iNOS, and TNF-α in *H. pylori*-infected Mongolian gerbils, protecting the gastric mucosa from *H. pylori* and the inflammation it causes [59].

#### 2.4.4. Soyasaponin Bb

Soyasaponin Bb (Figure 7D) is an oleanane-type pentacyclic triterpenoid saponin present in soybeans and other Fabaceae plants. As a principal component of soybean saponins in soybean seeds and processed products, soyasaponin Bb shows significant pharmacological effects including anti-inflammatory and anti-tumor properties [60]. In a diclofenac-induced rat GU model, soyasaponin Bb exhibited comparable protective effects as the positive control drug ranitidine. It elevated the levels of SOD and catalase (CAT), reduced the levels of MDA, TNF-α, and IL-6, while increasing the levels of PGE2 and gastric mucus secretion in the stomach. The reduction in inflammation by Soyasaponin Bb was connected to the inhibition of NF-κB-mediated regulation of COX-2 expression [61].

#### 2.4.5. δ-Amyrone

δ-Amyrone (Figure 7E), an oleanane-type pentacyclic triterpenoid from *Sedum lineare* Thunb., exhibits specific inhibitory activity against COX-2 and has no effect on COX-1, while also showing anti-inflammatory activity both in vitro and in vivo [62]. Pharmacological evaluation of δ-amyrone (4 and 8 mg/kg, p.o.) in ethanol-induced GU models revealed dose-dependent suppression of the expression of NF-κB, with subsequent attenuation of pro-inflammatory mediators (TNF-α, IL-6) and NO overproduction in gastric tissue. In addition, δ-amyrone elevated the gastric pH and gastric mucus content, inhibited the ethanol-induced increase in myeloperoxidase (MPO) levels, and significantly reduced gastric injury in mice [63].

#### 2.4.6. Maslinic Acid

Maslinic acid (Figure 7F) is an oleanane-type pentacyclic triterpenoid that is present in various medicinal and edible plants like loquat, patchouli, hawthorn, spinach, and eggplant. It exhibits a diverse range of biological activities, including antioxidant, anti-inflammatory, anti-diabetic, and anti-tumor [64]. Maslinic acid (10 mg/kg, p.o.), isolated from the methanol extract of *Plinia edulis* (Vell.) Sobral fruits through chromatographic column separation, demonstrated significant gastroprotective effects in both HCl/EtOH and indomethacin-induced GU models in mice. The ulcer area was reduced by 97.12% and 96.28%, respectively, in comparison to the control group. This anti-ulcer effect of maslinic acid is partially attributed to its prominent inhibitory effect on H^+^/K^+^-ATPase [65].

#### 2.4.7. α-Boswellic Acid

α-Boswellic acid (Figure 7G) is an oleanane-type pentacyclic triterpenoid mainly present in the resins secreted by plants of the Boswellia genus [66]. In an ethanol-induced GU model in rats, α-boswellic acid (200 mg/kg, p.o.) exerts gastroprotective effects by activating the nuclear factor erythroid 2-related factor 2/heme oxygenase-1 (Nrf2/HO-1) antioxidant pathway. Pretreatment with α-boswellic acid raised the pH of gastric juice, elevated gastric mucus secretion, increased the levels of PGE2 and NO as well as the activities of SOD and CAT, reduced MDA production and leukocyte infiltration, and markedly improved the morphology of gastric tissues in rats [67].

### 2.5. Ursane-Type Triterpenoids

#### 2.5.1. Ursolic Acid

**Figure 8:**
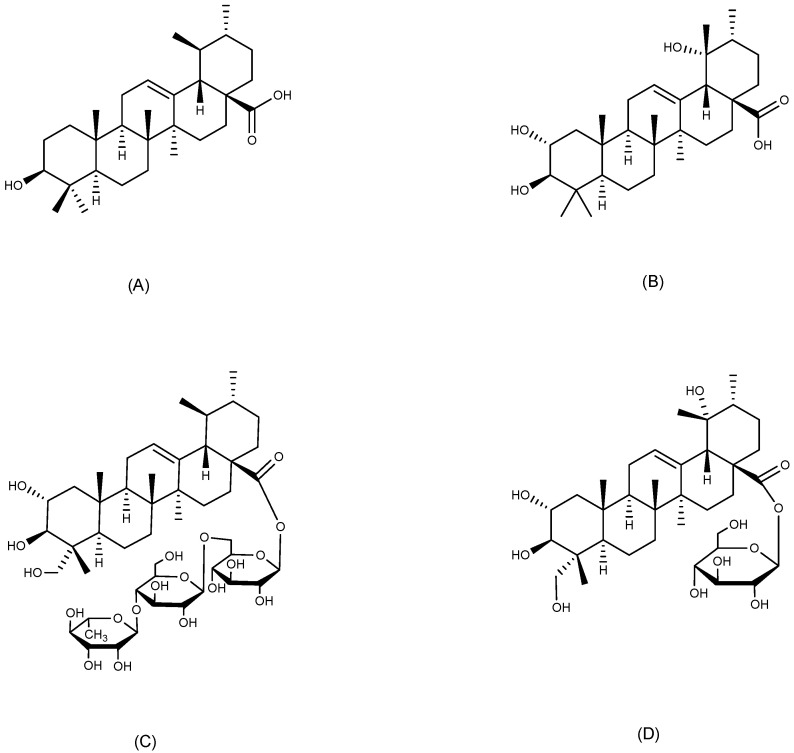
Ursane-type triterpenoids with anti-ulcer activity. (**A**) Ursolic acid; (**B**) Tormentic acid; (**C**) Asiaticoside; and (**D**) Niga-ichigoside F1.

Ursane-type compounds structurally derived from ursolic acid (Figure 8A), a phytochemical with common occurrence across the plant kingdom, can be found in apple peel, rosemary, and lavender [68]. Due to its multiple intracellular and extracellular targets, ursolic acid exhibits a wealth of pharmacological activities including antimetastatic, antioxidant, anti-inflammatory, and antimicrobial properties [69]. *Aganosma dichotoma* K. Schum is a plant traditionally utilized in Indian medicine for its anti-ulcer properties. Ursolic acid (50 mg/kg, p.o.) isolated from the ethanolic extract of its roots via high-performance thin-layer chromatography demonstrated a significant reduction in gastric juice volume and an elevation in gastric pH in pylorus-ligated rats. Additionally, ursolic acid exhibited anti-ulcer effects in both pylorus-ligated and ethanol-induced GU models in rats [70]. Pharmacological evaluation of ursolic acid derived from *Ochrosia elliptica* Labill. buds in an ethanol-induced GU model revealed that pretreatment with 100 mg/kg significantly reduced MDA levels and suppressed caspase-3 activity in gastric tissues compared to controls. These results indicate that the compound provides gastroprotective effects through dual mechanisms: attenuating mucosal oxidative stress and modulating apoptotic signaling pathways. Furthermore, the molecular docking results revealed that ursolic acid exhibits preferential binding affinity toward H^+^/K^+^-ATPase, demonstrating stronger molecular interactions than omeprazole and ranitidine, thereby highlighting its potential as a novel acid-suppressive therapeutic candidate [71].

#### 2.5.2. Tormentic Acid

Tormentic acid (Figure 8B) is a naturally occurring ursane-type pentacyclic triterpenoid, mainly extracted from the leaves and the whole herb of plants belonging to the Rosaceae family, though it is also present in the Labiatae and Urticaceae families. Tormentic acid has a multitude of pharmacological activities, including anti-inflammatory, anti-cancer, anti-diabetic, hepatoprotective, and neuroprotective [72]. The effects of tormentic acid on indomethacin-induced gastric injury were examined in vitro and in vivo. Tormentic acid (4 mg/kg) increased the levels of glutathione peroxidase (GSH-Px), SOD, and CAT, decreased the levels of MDA, TNF-α, IL-1β, and IL-6, elevated the expression of anti-inflammatory cytokines interleukin-4 (IL-4) and interleukin-10 (IL-10), and significantly reduced the area of ulcers in indomethacin-induced GU in rats. Additionally, tormentic acid inhibited indomethacin-induced apoptosis in human gastric mucosal epithelial cells (GES-1), while promoting cell proliferation and cell migration. The findings indicated that the gastric mucosal protective mechanism of tormentic acid was linked to the promotion of epithelial cell regeneration and migration, as well as the acceleration of gastric mucosal barrier remodeling [73].

#### 2.5.3. Asiaticoside

*Centella asiatica* (L.) Urban, a herbaceous perennial with pantropical distribution, holds dual ethnomedicinal significance in Ayurvedic and traditional Chinese medicine, particularly valued for its antipyretic properties and therapeutic efficacy in managing dermatological pathologies [74]. Asiaticoside (Figure 8C) is a natural ursane-type pentacyclic triterpenoid and a principal active ingredient of *Centella asiatica*. It has gained widespread attention in dermatological research due to its efficacy in wound healing and skin care. Moreover, asiaticoside possesses a variety of biological activities involving anti-inflammatory, neuroprotective, and anti-tumor activities [75]. In an acetic acid-induced GU model in rats, asiaticoside demonstrated the capacity to reduce MPO activity in ulcerated tissues, promote microangiogenesis at the base of the ulcer, and facilitate cell proliferation at the ulcer site, exhibiting anti-inflammatory, angiogenic, and cell proliferative properties [76]. In addition, asiaticoside dose-dependently attenuated the activity and protein expression of iNOS in the ulcerated tissues of rats with acetic acid-induced gastric injury, thereby inhibiting the overproduction of NO, which could aggravate the ulcers, and showing gastroprotective activity [77].

#### 2.5.4. Niga-Ichigoside F1

Niga-ichigoside F1(Figure 8D) is a natural ursane-type pentacyclic triterpenoid, predominantly distributed in the *Rubus* genus, which has been shown to have antioxidant, anti-inflammatory, and wound-healing properties [78,79]. In addition to these biological activities, there is a therapeutic effect of niga-ichigoside F1 on different types of GU. Niga-ichigoside F1 was isolated from the methanol extract of dried roots of *Rubus coreanus* Miq. via silica gel column chromatography. This compound significantly inhibited ethanol-salicylate-induced GU in rats and reduced gastric secretion, total acid excretion, and gastric juice acidity, while enhancing the activities of GSH-Px and SOD [80]. In a GU model in mice induced by HCl/EtOH, niga-ichigoside F1 (30 mg/kg) demonstrated anti-ulcer effects comparable to the positive control drug omeprazole, with an ulcer inhibition rate of 98.45%. This activity of niga-ichigoside F1 may be partly attributed to its anti-secretory effect [81].

### 2.6. Lupane-Type Triterpenoids

#### 2.6.1. Lupeol

**Figure 9:**
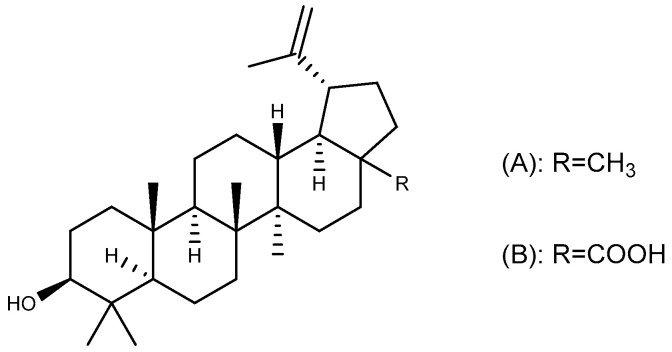
Lupane-type triterpenoids with anti-ulcer activity. (**A**) Lupeol and (**B**) Betulinic acid.

Lupeol (Figure 9A), a lupane-type pentacyclic triterpenoid widely distributed in nature, exhibits significant therapeutic potential across multiple domains. This compound exerts its effects through the modulation of critical pathological pathways including oxidative stress, inflammatory responses, and apoptotic regulation via diverse mechanisms. Consequently, it holds clinical relevance in the fields of oncology, metabolic disorders, cardiovascular diseases, and renal pathologies [82]. In an ethanol-induced GU model in mice, lupeol (30 mg/kg, p.o.) restored ethanol-depleted non-protein sulfhydryl groups (NP-SH) and exhibited stronger gastroprotective effects than the positive control drug N-acetylcysteine. This protective effect of lupeol was found to be attenuated by indomethacin and L-NAME, indicating that prostaglandins (PGs) and NO may play a role in its treatment [83]. Molecular interaction results revealed van der Waals interactions between lupeol and key amino acid residues of gastric H^+^/K^+^-ATPase, suggesting that this compound has great potential as an anti-ulcer agent through anti-secretory effects [84].

#### 2.6.2. Betulinic Acid

Betulinic acid (Figure 9B) is a lupane-type pentacyclic triterpenoid commonly found in the bark of *Betula platyphylla* Suk. and has therapeutic effects on tumors, inflammation, and diabetes [85]. In an indomethacin-induced GU model, the ulcer index of rats pretreated with betulinic acid for seven consecutive days was significantly reduced in a dose-dependent manner compared with the control group. This anti-ulcer effect of betulinic acid is attributed to an increase in the number of gastric mucus cells and gastric mucus secretion, as well as a decrease in gastric acid secretion and MDA level in the stomach [86].

### 2.7. Friedelane-Type Triterpenoid

**Figure 10:**
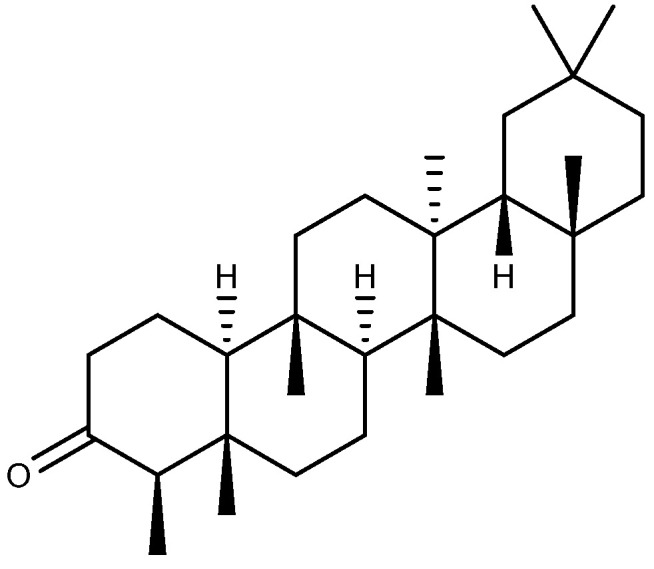
The structure of friedelin.

Friedelin (Figure 10), a friedelane-type pentacyclic triterpenoid, is commonly identified in the bark and leaves of diverse plant species. In addition to being used as an insecticide and herbicide, friedelin has applications in the treatment of cancer, diabetes, inflammation, and neurological disorders [87]. Friedelin (35 mg/kg, p.o.) significantly inhibited indomethacin-induced GU in rats. Apart from exerting anti-inflammatory and antioxidant effects by regulating the levels of glutathione (GSH), MPO, MDA, TNF-α, and IL-10, friedelin can also increase the volume of gastric mucus and raise the pH of gastric juice, as well as downregulate the level of caspase-3 [88].

### 2.8. Other Triterpenoids

**Figure 11:**
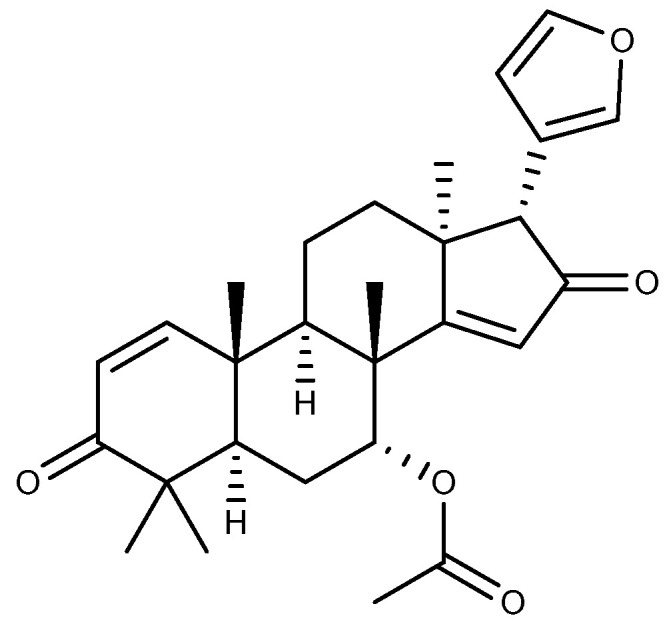
The structure of azadiradione.

Azadiradione (Figure 11) belongs to the limonoids and serves as an active ingredient in the insecticidal plant *Azadirachta indica* A. Juss., which is mainly distributed in the tropical region of southeastern Asia [89]. In addition to its insecticidal properties, azadiradione (20 and 40 mg/kg, p.o.) isolated from the ethanol extract of *Azadirachta indica* seeds via silica gel column chromatography demonstrated significant protective effects in cold restraint stress, ethanol, pyloric ligation, and aspirin-induced GU in rats. The anti-ulcer effect of azadiradione was superior to that of the positive control drug sucralfate in an ethanol-induced ulcer model. This anti-ulcer property is related to the promotion of gastric mucin secretion, the inhibition of H^+^/K^+^-ATPase, and the increase in PGE2 levels in the stomach [90].

The effects of the above triterpenoids on GU are summarized and shown in Table 1.

## 3. The Pharmacological Mechanisms of Triterpenoids in GU

### 3.1. Regulation of Redox Balance

The equilibrium between oxidative stress and antioxidant defenses within the gastric mucosa governs both the initiation and progression of GU fundamentally. Reactive Oxygen Species (ROS) are a group of unstable molecules produced by various cells, which include hydrogen peroxide (H_2_O_2_), hydroxyl radicals (OH^−^), singlet oxygen (^1^O_2_), and superoxide (O_2_^−^). These substances are generally considered to be harmful to the body [91]. Excessive ROS are generated when the body is exposed to stimuli such as ischemia, hypoxia, and radiation. These ROS can damage membranes and cellular macromolecules, induce apoptosis, and lead to gastric injury [92]. Furthermore, MPO located in neutrophils will also produce excessive ROS when neutrophils are activated. Subsequently, ROS react with H_2_O_2_ to produce large quantities of HOCl. HOCl will react vigorously with sulfhydryl groups and deplete GSH in significant quantities, thereby reducing the body’s antioxidant capacity [93]. GSH contains a readily oxidizable sulfhydryl group that reacts with ROS to eliminate it. GSH also assists GSH-Px in reducing H_2_O_2_ to H_2_O. The antioxidant enzyme system in cells is composed of GSH-Px, CAT, and SOD. This system can effectively scavenge ROS while modulating systemic redox homeostasis [94]. These bioactive components coordinately regulate redox equilibrium through synergistic interactions. The balance is disrupted when there is a significant alteration in the levels of these substances in the stomach, thereby leading to oxidative stress. Triterpenoids such as tormentic acid [73], asiaticoside [76], and niga-ichigoside F1 [80] can enhance the activities of GSH-Px, CAT, and SOD, increase the level of GSH, and reduce the activity of MPO, suggesting that they are able to protect the gastric mucosa from oxidative damage.

GSH, as a key constituent of NP-SH, critically orchestrates GU pathophysiology through mucosal defense mechanisms. Because of its reduced sulfhydryl group, NP-SH is capable of binding to free radicals produced by ulcerogenic agents, thereby exhibiting antioxidant effects [95]. Meanwhile, NP-SH enhances the disulfide bonds between gastric mucus subunits, thus reinforcing the gastric mucus barrier and safeguarding the gastric mucosa from invasive factors [96,97]. Lupeol [83] restores ethanol-depleted NP-SH levels that prevent the exacerbation of GU.

Nrf2 also plays a significant role in clearing the oxidative stress state of gastric tissues. Under oxidative stress, cytoplasmic Nrf2 translocates to the nucleus, which in turn upregulates the expression of key antioxidant enzymes including SOD, CAT, and GSH-Px. The activation of Nrf2 also protects tight junctions, promotes gastric mucus secretion, and downregulates NF-κB to reduce inflammatory response [98,99]. In addition, HO-1, an antioxidant factor regulated by Nrf2, protects cells and inhibits ROS production, thereby protecting the gastrointestinal tract from oxidative stress damage [4,100]. The triterpenoid α-boswellic acid [67] can upregulate the expression of Nrf2, which exhibits antioxidant effects.

### 3.2. Regulation of Inflammatory Cytokines

The inflammatory reaction of gastric mucosa caused by various stimuli is a major cause of the occurrence and development of GU. Macrophages, as key cells in human innate immunity, are pivotal in mediating the inflammatory response. Once activated by different stimuli, macrophages form two phenotypes, M1 and M2, with pro-inflammatory and anti-inflammatory activities, respectively [101]. Macrophage activation by lipopolysaccharide and Th1 cytokines (including TNF-α) leads to the formation of M1 macrophages, which are capable of releasing cytokines such as TNF-α, IL-1β, and IL-6. In contrast, M2 macrophages activated by Th2 cytokines release cytokines such as IL-10 [102]. Thus, the pro-inflammatory factors TNF-α, IL-1β, and IL-6 contribute to the progression of GU, whereas the anti-inflammatory factor IL-10 reduces the severity of inflammation in GU [103]. Tormentic acid [73] and friedelin [88] are able to modulate the levels of these cytokines in different ulcer models to protect the gastric mucosa from inflammatory damage.

As a member of the CC chemokine family, MCP-1, alternatively designated C-C motif chemokine ligand 2, functions as a low-molecular-weight cytokine involved in immune regulation. It serves a pivotal function in the process of inflammation by regulating the migration and infiltration of inflammatory cells at the site of inflammation [104]. Previous research has indicated the involvement of MCP-1 in the formation of ulcers. The administration of TNF-α results in an increase in MCP-1 expression, which in turn regulates leukocyte recruitment and ultimately contributes to the recurrence of GU [105]. Astragaloside IV [41] reduced the levels of TNF-α and MCP-1 and exhibited anti-inflammatory effects in an acute GU model in rats. LTB4 is also associated with the promotion of leukocyte migration into inflamed tissues. When LTB4 is overproduced, it becomes involved in the production and persistence of inflammation [106]. A previous study demonstrated that indomethacin resulted in a significant elevation in gastric LTB4 levels and leukocyte infiltration in mice, which subsequently triggered an inflammatory response and gastric damage [107], whereas 3α-hydroxymasticadienoic acid [45] can reduce the level of LTB4 in the stomach and leukocyte infiltration, thereby inhibiting the inflammation of gastric mucosa.

NF-κB can activate the expression of genes associated with inflammation, thereby exerting a vital influence on the inflammatory process [108]. In response to stimulation by pro-inflammatory factors or ROS, activation of the transcriptional regulator NF-κB triggers downstream inflammatory pathways, elevating levels of cytokines including TNF-α, IL-1β, and IL-6, which collectively amplify GU progression [109,110]. The NF-κB pathway was inhibited and the levels of inflammation-associated cytokines were modulated in different ulcer models by ginsenoside Rh4 [31], soyasaponin B [61], and δ-amyrone [63]. This indicates that these triterpenoids protect gastric tissue by reducing the inflammation associated with ulcerative lesions.

ET-1, a powerful vasoconstrictor present in the gastrointestinal tract, is also a potent ulcerogenic agent. Excessive release of ET-1 due to various reasons can dramatically lower gastric blood flow and dysregulate microcirculation of the gastric mucosa, ultimately leading to gastric mucosal damage [111,112]. It has been shown that ET-1 significantly delays ulcer healing and scar formation in established GU [113]. ET-1 can also increase the expression of TNF-α, IL-6, and other inflammatory factors, which is a positive feedback process. As a result, these factors will in turn stimulate the synthesis and release of ET-1, worsening the inflammatory response [114]. Triterpenoids such as ginsenoside Rg3 [28], protopanaxatriol [34], and ocotillol [37] exhibit gastroprotective effects by decreasing ET-1 levels in a variety of GU models.

### 3.3. Regulation of Gastric Mucosal Cytoprotective Factor

In the presence of gastric acid, pepsin, and foreign invading factors, the maintenance of gastric mucosal tissue structure and normal physiological functions cannot be achieved without the support of cytoprotective factors. Cytoprotective factor enhances gastric mucus secretion while maintaining the integrity of the gastric epithelium, thereby reinforcing the defensive capabilities of the gastric mucosa and mitigating the damage associated with GU [115,116]. Among these protective factors, PGE2, NO, H_2_S, and Hsp 70 play a pivotal role in the inhibition of GU by triterpenoids.

Endogenous PGs constitute a class of lipid mediators produced from arachidonic acid via prostaglandin synthase and two isomers of cyclooxygenase (COX-1 and COX-2). They possess potent gastrointestinal protective properties [117]. In normal gastric mucosa, COX-1 is highly expressed, while COX-2 is almost unexpressed. However, the expression level of COX-2 is increased in the presence of inflammation or tissue injury [118]. Among these endogenous PGs, the role of PGE2 is the most important. The classification of PGE2 receptors into four distinct subtypes provides further evidence that PGE2 exerts a multitude of effects on the gastrointestinal tract. Through the activation of various receptors, PGE2 is capable of increasing gastric mucus and HCO_3_^−^ secretion, inhibiting gastric acid secretion and gastric motility, enhancing gastric mucosal blood flow, and impeding neutrophil migration [119,120]. A number of triterpenes, including ginsenoside Rh4 [31], astragaloside IV [42], and friedelin [88], have been demonstrated to enhance the gastric mucosal barrier by regulating the activity of COX and the level of PGs.

The balance of the gastric environment is influenced by the presence of certain gaseous media. NO is a small molecule gaseous mediator produced by the enzyme nitric oxide synthase (NOS). Its effects on the health of the gastric mucosa are double-edged. NOS is categorized into three subtypes, namely neuronal NOS (nNOS), endothelial (eNOS), and iNOS. eNOS and nNOS are integral to gastric homeostasis. NO produced by them can mediate gastric mucosal blood flow, decrease gastric acid secretion by regulating endogenous PGs, attenuate oxidative stress through free radical scavenging, and sustain goblet cell secretory activity for mucus barrier integrity [121,122]. Triterpenoids such as astragaloside IV [42] and α-boswellic acid [67] upregulate the NO level. Nevertheless, there is a strong correlation between iNOS and the severity of inflammation. Pro-inflammatory cytokines, including TNF-α and IL-1β, facilitate the expression of iNOS and consequently result in a significant increase in NO production. Excessive amounts of NO were found to disrupt mitochondrial respiration, damage DNA, promote mutation, and ultimately induce apoptosis and exacerbate inflammation [123,124]. Ginsenoside Rg3 [28] and asiaticoside [77] could protect gastric mucosa from the negative effects of NO by blocking iNOS.

In addition to upregulating NO levels, 3α-hydroxymasticadienoic acid [45] increased the levels of another gaseous mediator, H_2_S, in the stomach. H_2_S can activate mitogen-activated protein kinase and Nrf2, thereby enhancing cellular antioxidant capacity to counteract oxidative injury in the gastric mucosa. Furthermore, H_2_S reduces gastric acid secretion and increases the level of HCO_3_^−^ in the stomach, a process that is mediated by NO and PGs. This ultimately protects the gastric mucosa from damage [125].

External stimuli, such as oxidative stress, lead to an increase in the levels of Hsp 70, a member of the heat shock family of proteins that is widely found in mammalian cells. Hsp 70 removes damaged denatured proteins to preserve the functional structure of tissue proteins, thus preventing apoptosis [126]. In addition, Hsp 70 enhances the tightness of cell junctions, thereby safeguarding cells from injury [127]. Several studies have demonstrated that elevated levels of Hsp 70 exert a beneficial influence on gastric mucosal protection [128,129]. Astragaloside IV [41] can regulate the level of Hsp 70 in GU models, enhance the protective effect of gastric mucosa, and promote the healing of gastric mucosal injury.

### 3.4. Regulation of the Acidity and Viscosity of Gastric Juice

Digestion is one of the main functions of the stomach. The production of gastric acid is a crucial element of this process, yet it is also a contributing factor to the formation of GU [130]. Gastric parietal cells exchange H^+^ from the cytoplasm to the gastric lumen through H^+^/K^+^-ATPase, and H^+^ combines with Cl^−^ in the gastric lumen to form gastric acid. The secretion process of gastric acid is modulated through the nervous system, hormones, and paracrine secretion. Exogenous factors may induce the dysregulation of secretion, which triggers erosive effects on gastric mucosa [130,131]. Triterpenoids including maslinic acid [65] and ursolic acid [71] inhibit H^+^/K^+^-ATPase, which in turn exhibits anti-secretory effects.

The gastric mucus covering the gastric epithelial cells represents the initial line of defense for the gastric tissue. Gastric mucus is primarily composed of water, glycolipids, proteoglycans, and glycoproteins. Among these components, mucin, a highly glycosylated glycoprotein secreted by goblet cells, serves as a crucial component of the mucus layer [132]. Mucus is hydrophilic, and its capacity to bind water serves as a diffusion barrier that protects gastric epithelial cells from gastric acid and proteases, thereby preventing self-digestion [133]. Gastric mucus plays an indispensable role in gastric healing by acting as a barrier between the stomach and the external environment during the regeneration of epithelial cells [134]. The level of gastric mucus and mucin is closely related to the generation and healing of GU. Ginsenoside Rb1 [26], oleanolic acid [49], and betulinic acid [86] all increase gastric mucus or mucin levels, suggesting that these triterpenoids may exert gastroprotective and ulcer healing effects by enhancing the gastric mucosal barrier.

### 3.5. Inhibition of Apoptosis

Apoptosis constitutes a core pathomechanism in GU development. In mammalian cells, apoptosis is subdivided into two canonical pathways: the intrinsic or mitochondrial pathway and the extrinsic or death receptor pathway [135]. Stimuli such as ROS can trigger the intrinsic pathway of apoptosis, which activates the pro-apoptotic protein Bax of the Bcl-2 family. Bax inhibits the expression of the anti-apoptotic protein Bcl-2 on the mitochondrial membrane, resulting in the opening of the mitochondrial membrane channels. Subsequently, the pro-apoptotic factor cytochrome c is released, which then activates caspase-9 to interact with caspase-3, ultimately leading to apoptotic cell death [4,136,137]. The induction of apoptosis through Fas, on the other hand, belongs to the extrinsic or death receptor pathway. Fas is a death receptor that is situated on the cell surface. When it is combined with the Fas ligand (FasL), a transmembrane protein belonging to the TNF family, Fas activates caspase-8 to interact with caspase-3, thereby inducing cell apoptosis [137,138]. Some members of the caspase family, such as caspase-1, contribute to the process of inflammation. Apoptosis or inflammatory factors can activate caspase-1, and activated caspase-1 can exacerbate inflammation through a positive feedback mechanism [137]. Triterpenoids, including ginsenoside Rh4 [31], araloside A [53], and friedelin [88], have been demonstrated to regulate the levels of the aforementioned factors, thereby protecting gastric tissue from apoptosis and inflammatory destruction.

### 3.6. Inhibition of Helicobacter Pylori

*H. pylori* is a Gram-negative bacterium that can be transmitted via fecal–oral and oral–oral routes. In numerous developing countries, the infection rate exceeds 50%, making it a major epidemiological driver of the prevalence and progression of GU [139]. *H. pylori* can produce a substantial quantity of urease, which catalyzes the hydrolysis of urea to ammonia and carbonic acid. As a result, gastric acid is neutralized, creating a nearly neutral microenvironment that supports the survival of Helicobacter pylori in the stomach. Simultaneously, this process alters the gastric mucus layer from a gel to a viscous solution, thereby enabling *H. pylori* to utilize its flagella to traverse the gastric mucus layer and attach to gastric epithelial cells [140]. *H. pylori* can induce morphological changes in host cells by expressing virulence genes, resulting in an inflammatory response and the promotion of apoptosis [141,142]. 18β-Glycyrrhetinic acid [59] demonstrates inhibitory effects on *H. pylori*, elevates gastric juice pH, and attenuates the inflammatory response induced by *H. pylori*. These findings suggest that triterpenoids may protect gastric tissues via the inhibition of *H. pylori*.

### 3.7. Promotion of GU Healing

**Figure 12:**
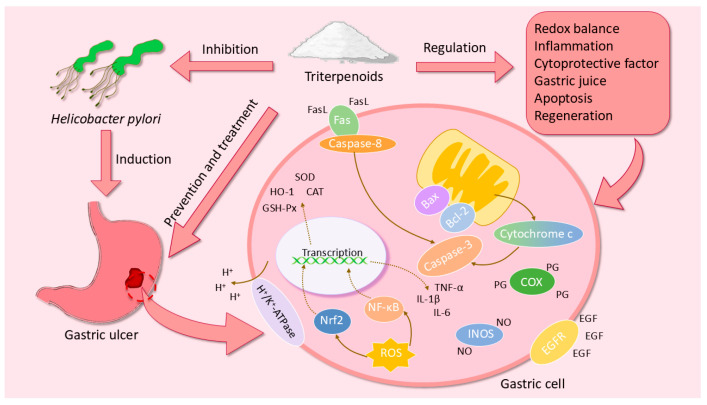
The pharmacological mechanisms of triterpenoids in the prevention and treatment of GU. Triterpenoids can prevent and treat GU through various pathways, such as the redox balance, inflammation, cytoprotective factor, gastric juice, apoptosis, regeneration, and antibacterial pathways.

The promotion of ulcer healing represents a crucial aspect of the mechanism through which triterpenoids exert their anti-ulcer effects. These effects are linked to a multitude of growth factors, including epidermal growth factor (EGF) and epidermal growth factor receptor (EGFR). EGF is a polypeptide that inhibits gastric acid secretion and promotes the proliferation and differentiation of mucosal epithelial cells [143]. In addition to its direct interaction with EGFR, EGF can also activate COX-2 to produce PGE2, which indirectly activates EGFR through matrix metalloproteinases, thereby accelerating the healing of GU [144]. Triterpenoids, including ginsenoside Rg3 [28], protopanaxatriol [34], and ocotillol [37], are capable of elevating the levels of EGF and EGFR in diverse GU models, exhibiting a significant effect on the promotion of ulcer healing. The pharmacological mechanisms of triterpenoids in the prevention and treatment of GU are shown in Figure 12.

## 4. The Animal Models Employed in Research on Triterpenoids in GU

**Figure 13:**
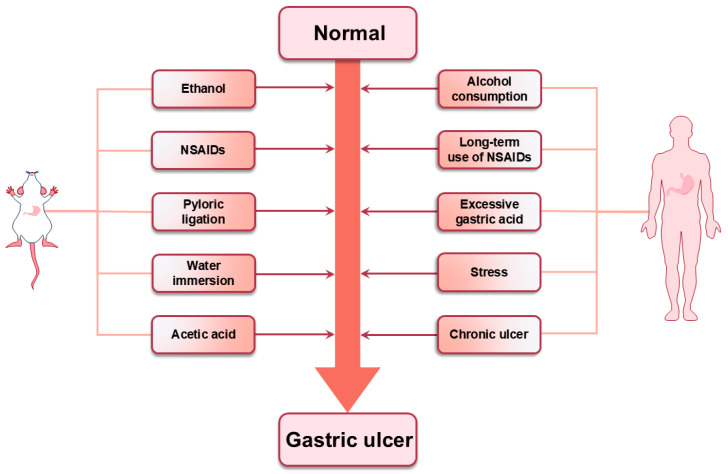
The relationship between experimental animal models of GU and human GU. Previous studies utilized diverse GU models, including ethanol, NSAIDs, pylorus ligation, restraint water-immersion, and acetic acid-induced GU models. These models, respectively, correspond to alcohol consumption, long-term NSAID use, excessive gastric acid, stress-induced GUs, and chronic GU.

Triterpenoids isolated from natural medicines can protect gastric mucosa from both endogenous and exogenous invasive factors. This is achieved through a range of complex and diverse mechanisms of action. The discovery of these triterpenoids with pronounced pharmacological effects could not be achieved without the support of animal models. The utilization of experimental animal models has facilitated a more profound comprehension of the etiology, pathology, and overall characteristics of GU in humans. Previous studies have used a variety of GU models, including ethanol, NSAIDs, pylorus ligation, and acetic acid-induced GU models. These models are inextricably linked to the triggers that produce GU in humans (Figure 13). Understanding the pathophysiological mechanisms by which they induce ulcers can help to better address the challenges posed by GU.

Alcohol is one of the most widely used addictive substances. The excessive intake of alcohol may result in the development of GU. Consequently, ethanol has been extensively employed as an ulcerogenic agent in ulcer models [145]. Ethanol exposure induces structural and functional perturbations in the gastric mucosal barrier, characterized by the depletion of endogenous antioxidant systems (SOD and GSH) coupled with the enhanced generation of ROS. This impairs the antioxidant capacity of the organism and ultimately gives rise to oxidative stress and apoptosis [145,146]. The levels of pro-inflammatory factors such as NF-κB, TNF-α, and IL-6, which trigger inflammation and slow ulcer healing, also increase with ethanol consumption [145,147]. Ethanol inhibits the activity of acetaldehyde dehydrogenase, leading to the accumulation of acetaldehyde in the stomach. This accumulation of acetaldehyde induces the death of gastric mucosal cells and destroys mitochondria, resulting in the release of ROS and subsequent oxidative stress [148,149]. Furthermore, ethanol can also impede the secretion of PG in the stomach, which in turn damages the microcirculation of the gastric mucosa and inhibits gastric mucosal regeneration [146]. Ginsenoside Rd [27], astragaloside IV [40], and α-boswellic acid [67] have been demonstrated to inhibit the development of ethanol-induced GU in rats. The effects of these triterpenoids can be attributed to the attenuation of ethanol-induced oxidative stress and inflammation.

NSAIDs have become among the most widely used over-the-counter drugs globally due to their efficacious antipyretic, analgesic, and anti-inflammatory properties [150,151]. Nevertheless, the prolonged use of NSAIDs frequently results in gastric mucosal erosion, gastrointestinal bleeding, gastric perforation, and GU. Approximately 13% of patients with GU are associated with the use of NSAIDs, with indomethacin often employed in animal models of GU due to its propensity to induce gastric injury [151,152]. NSAIDs primarily exert anti-inflammatory effects by inhibiting the two subtypes of COX, COX-1 and COX-2. Unfortunately, this is also one of the mechanisms by which NSAIDs induce GU. This inhibition of COX reduces the body’s PG level, decreases mucosal blood flow, decreases gastric mucus and bicarbonate secretion, and ultimately leads to gastric mucosal damage [152,153,154]. NSAIDs combine with the phospholipids of gastric mucosal epithelial cells, destroying their hydrophobic properties and increasing the permeability of the gastric mucosa. This results in the diffusion of gastric acid into the gastric mucosal barrier, which in turn leads to cellular necrosis and the induction of GU [151,153]. The triterpenoids 3α-hydroxymasticadienoic acid [45], astragaloside IV [42], and betulinic acid [86] exhibit the ability to safeguard gastric mucosa from NSAID-induced damage. This is achieved through the upregulation of inhibited COX activity, the increase in prostaglandin secretion, and the enhancement of the gastric mucosal barrier function. Consequently, these triterpenoids can be employed in conjunction with NSAIDs to mitigate the gastrointestinal adverse effects associated with NSAID treatment and enhance therapeutic efficacy.

In addition to the utilization of pharmacological agents to induce GU, the pyloric ligation model represents a principal methodology in experimental gastroenterology research. This method results in the excessive accumulation of gastric acid and the destruction of the gastric mucosa, which ultimately leads to the formation of GU [155]. Following pyloric obstruction caused by pyloric ligation, the vago-vagal reflex is activated by mechanoreceptors within the pyloric region, which stimulates the secretion of gastric acid through the neuronal, endocrine, and paracrine pathways, leading to gastric acid accumulation [156,157]. The excessive production of gastric acid not only alters the permeability of the gastric mucosa but also stimulates the secretion of pepsin, which facilitates the self-digestion of the gastric mucosa. This process ultimately leads to the destruction of the gastric mucosa and the acceleration of the ulceration process [158,159]. Additionally, pyloric ligation-induced GU has been demonstrated to be linked to oxidative stress, a process that stimulates ROS production and ultimately results in decreased SOD and GSH levels and increased MDA levels [157,159]. Ginsenoside Rg3 [28], araloside A [52], and azadiradione [90] can exhibit inhibitory effects on pyloric ligation-induced GU by reducing gastric acid production and inhibiting oxidative stress.

Stress is a major cause of GU [160]. The use of stress models for research can better clarify the treatment direction of stress ulcers. Stress stimulates the sympathetic and parasympathetic nerves, increases gastric motility and the contraction of the gastric muscles, and constricts small arterial vessels. This results in the compression of gastric blood vessels and a reduction in gastric blood flow, which in turn causes mucosal hypoxia and ischemia. In this state, a substantial quantity of ROS is produced, which continues to activate the NF-κB-mediated inflammatory pathway. As a result, oxidative stress and inflammation are triggered, ultimately leading to the development of GU [161,162]. In addition, stress also results in the overproduction of NO within the body, leading to a reduction in the activity of antioxidant enzymes, thus weakening the defense of the gastric mucosa [163]. Triterpenoids, including astragaloside IV [41], araloside A [52], and azadiradione [90], demonstrated anti-ulcer effects in this model. The therapeutic efficacy may derive from multimodal actions involving inflammatory response modulation, oxidative stress attenuation, and the enhancement of the gastric mucosal defense.

In contrast to the acute ulcer model described above, the GU model induced by acetic acid is a chronic ulcer model. The acetic acid-induced GU model exhibits high clinical relevance by accurately mirroring both the histological architecture and healing process characteristic of human chronic ulcers. In consequence, this model is often used to assess the anti-ulcer properties of drugs [164]. Acetic acid reduces the levels of prostaglandins, growth factors, and NO in the stomach. Additionally, emerging evidence implicates it in the induction of pro-inflammatory cytokine upregulation alongside altering the microcirculation and adhesion patterns of gastric mucus, culminating in the structural and functional compromise of gastric mucosa. This damage penetrates deeply into the muscularis propria, leading to the formation of chronic GU within 2–3 days [164,165,166]. Ocotillol [37], oleanolic acid [49], and asiaticoside [76] exhibit anti-ulcer and healing-promoting properties in this model by enhancing gastric mucosal defenses, exerting anti-inflammatory effects and antioxidant effects.

## 5. The Current Limitations and Challenges of Triterpenoids in GU

Although triterpenoids demonstrate promising therapeutic potential for GU, several critical challenges limit their advancement as bioactive components. 18β-Glycyrrhetinic acid exhibits strong antibacterial effects against *H. pylori*, suggesting its value in protecting gastrointestinal health. However, a report reveals that prolonged high-dose oral administration may induce pseudoaldosteronism, manifesting as hypertension, hypokalemia, and metabolic alkalosis [167]. Ginseng, a widely used medicinal plant, contains triterpenoids as its primary active components with multiple gastroprotective properties. Notably, ginsenoside Rb1 demonstrates embryotoxicity and teratogenic effects in rodent whole-embryo culture experiments [168]. High oral doses (600 mg/kg) of rare ginsenosides like Rh4 altered gut microbiota composition and disrupted the metabolism pathways of vitamin B6, glutathione, arginine, and proline in rats, ultimately causing liver injury [169]. These compounds also face bioavailability challenges. Their lipophilic nature and large molecular size restrict absorption, and astragaloside IV shows bioavailability of only 7.4% and 3.66% in dogs and rats, respectively [38]. Most dammarane-type triterpenoids exhibit similarly low absorption rates. The mean half-lives of ginsenoside Rb1 and ginsenoside Rd in plasma were 17.96 h and 19.29 h, respectively, indicating that these triterpenoids could be rapidly metabolized [170]. Furthermore, clinical evidence remains insufficient. Current research predominantly relies on animal models and lacks large-scale human trials to comprehensively validate the safety and efficacy of triterpenoids for GU treatment. These combined limitations (safety concerns, pharmacokinetic challenges, and inadequate clinical verification) constrain their therapeutic application potential.

Nevertheless, emerging evidence highlights promising avenues for improvement. Certain triterpenoids exhibit effective anti-ulcer doses significantly below toxic thresholds. Ursolic acid shows a mouse acute LD50 of 9.26 g/kg [171], far surpassing its effective dose for GU treatment (100 mg/kg). Notably, this compound has been safely consumed as a dietary supplement without reported adverse events [172]. Friedelin exerts significant anti-ulcer effects at 35 mg/kg, with the oral administration of 80 mg/kg in rats showing no behavioral alterations or mortality within 24 h [88]. Bioavailability enhancement strategies show particular promise. Structural modifications of free triterpenoids can improve water solubility and biological activity [50,173], facilitating drug delivery. Advanced formulations like nano-formulations and gastroretentive delivery systems enhance gastric targeting and prolong therapeutic action [174]. Combination therapy with absorption enhancers (e.g., piperine) significantly increases triterpenoid permeability and bioavailability [175]. These findings emphasize the urgent need to prioritize translational research. Subsequent investigations should focus on comprehensive long-term toxicological evaluations to establish safety profiles for GU treatment applications. Such approaches will generate scientifically robust evidence to guide clinical development, ultimately bridging the gap between laboratory discoveries and practical therapeutic implementations.

## 6. Conclusions

In summary, triterpenoids isolated from natural medicines can treat and prevent GU induced by a variety of factors, including alcohol, NSAIDs, *H. pylori*, and stress. This is achieved through a range of mechanisms, including anti-inflammatory, antioxidant, cytoprotective, anti-secretory, anti-apoptotic, antibacterial, and ulcer-healing promotion. The rise in drug abuse and *H. pylori* resistance highlights the necessity of the development of more efficacious treatment options and superior pharmaceutical agents to address these challenges. With their recognized safety, reliability, and favorable biological activities, triterpenoids isolated from natural medicines have the potential to be utilized as dietary supplements or adjuvants to the prevention and treatment of GU as well as to lessen side effects that other medications may cause. It is anticipated that these compounds will emerge as one of the candidates for anti-ulcer drugs in the future.

## Figures and Tables

**Table 1:** Triterpenoids with anti-ulcer activity.

Compound	Plant Source	Type	Model	Efficacy	Effect or Mechanism	Ref.
Ginsenoside Rb1	*Panax ginseng* C. A. Mey.	Dammarane	Male SD rats (oral EtOH/HCl (60%/150 mM, 5 mL/kg))	60.6% ^1^ (300 mg/kg)	↑ mucus	[26]
Ginsenoside Rd	*Panax ginseng* C. A. Mey.	Dammarane	Rats (oral ethanol (20 mg/kg))	57.6% ^2^ (100 mg/kg)		[27]
			Rats (oral indomethacin (20 mg/kg))	52.1% ^2^ (100 mg/kg)		
Ginsenoside Rg3	*Panax ginseng* C. A. Mey.	Dammarane	Male Wistar rats (oral ethanol (5 mg/kg))	63.1% ^1^ (20 mg/kg)	↑ SOD, NO, EGF, EGFR ↓ iNOS, ET-1	[28]
			Male Wistar rats (pyloric ligation)	64.8% ^1^ (20 mg/kg)		
			Male Wistar rats (inject 0.3 mL indomethacin)	62.7% ^1^ (20 mg/kg)		
Ginsenoside Rh4	*Panax notoginsen* (Burkill) F. H. Chen ex C. Y. Wu & K. M. Feng	Dammarane	Male SD rats (absolute ethanol (5 mg/kg))	92.57% ^2^ (60 mg/kg)	↑ NO, PGE2, COX-2, Bcl-2 ↓ MAPK/NF-κB signaling pathway, Bax, Fas	[31]
Protopanaxatriol	*Panax ginseng* C. A. Mey.	Dammarane	Male Wistar rats (inject 0.3 mL indomethacin)		↑ SOD, EGF, EGFR ↓ TNF-α, IL-6, ET-1, MDA	[34]
Ocotillol	Panax plants	Ocotillol	Male Wistar rats (inject 0.3 mL indomethacin)		↑ NO, SOD, EGF, EGFR ↓ ET-1	[37]
Astragaloside IV	*Astragalus membranaceus* (Fisch.) Bunge	Cycloartane	Male Wistar rats (oral absolute ethanol (1 mg/rat))	52.3% ^2^ (30 mg/kg)	↑ NO	[40]
			Male SD rats (water immersion and restraint stress)	70.79% ^2^ (50 mg/kg)	↑ PH, mucus, SOD, HSP70 ↓ MDA, TNF-α, MCP1	[41]
			Wistar rats (oral aspirin (150 mg/kg))		↑ COX-1, PGE2, NO, SOD	[42]
3α-Hydroxymasticadienoic acid	*Amphipterygium adstringens* (Schltdl.) Schiede ex Standl	Tirucallane	Male Wistar rats (oral indomethacin (30 mg/kg))	70% ^2^ (30 mg/kg)	↑ SOD, PGE2, NO, H_2_S ↓ TNF-α, LTB4	[45]
Oleanolic acid	Oleaceae plants	Oleanane	Male SD rats (inject 0.05 mL of 30% acetic acid)	76.0% ^2^ (100 mg/kg)	↑ mucus	[49]
			AGS (10 mM NaT for 30 min)		↑ PGE2	[50]
Araloside A	Aralia plants	Oleanane	Male SD rats (oral EtOH/HCl (60%/150 mM, 1.5 mL/rat))	51.4% ^1^ (100 mg/kg)	↑ pH	[52]
			Male SD rats (aspirin (100 mg/kg))	80.7% ^1^ (100 mg/kg)		
			Male SD rats (water immersion stress)	84.3% ^1^ (100 mg/kg)		
			Male SD rats (pyloric ligation)	73.9% ^1^ (100 mg/kg)		
			Male Kunming mice (oral 80% ethanol containing 15 mg/mL aspirin (10.0 mL/kg))	About 40% ^1^ (40 mg/kg)	↑ pH, mucus, Bcl-2 ↓ H^+^/K^+^-ATPase, cytochrome c, caspase-3, caspase-9, Bax	[53]
Soyasaponin Bb	Fabaceae plants	Oleanane	Male Wistar albino rats (diclofenac sodium (5 mg/mL))		↑ PGE2, mucus, CAT, SOD ↓ COX-2, MDA, TNF-α, IL-6, NF-κB	[61]
δ-Amyrone	*Sedum lineare* Thunb.	Oleanane	Male Kunming mice (oral 75% ethanol (0.5 mL/100 g))		↑ pH, mucus ↓ MPO, TNF-α, IL-6, NO, NF-κB	[63]
Maslinic acid	Loquat, patchouli, hawthorn, spinach, and eggplant	Oleanane	Female Swiss mice (oral EtOH/HCl (60%/0.3 M, 10 mL/kg))	97.12% ^2^ (10 mg/kg)	↓ H^+^/K^+^-ATPase	[65]
			Female Swiss mice (oral indomethacin (80 mg/kg))	96.28% ^2^ (10 mg/kg)		
α-Boswellic acid	Boswellia plants	Oleanane	Male SD rats (oral absolute ethanol (5 mL/kg))	42.45% ^1^ (200 mg/kg)	↑ pH, mucus, PGE2, NO, CAT, SOD, Nrf 2/HO-1 ↓ MDA	[67]
Ursolic acid	Apple peel, rosemary, and lavender	Ursane	Female Wistar albino rats (oral 95% ethanol (1 mL/rat))	96.9% ^1^ (100 mg/kg)	↓ MDA, caspase-3, H^+^/K^+^-ATPase	[71]
Tormentic acid	Rosaceae plants	Ursane	Male SD rats (oral indomethacin (100 mg/kg))		↑ GSH-Px, SOD, CAT, IL-10 ↓ MDA, TNF-a, IL-1b, IL-6, IL-4	[73]
			GES-1 (700 μM indomethacin for 18 h)		↑ cell migration ↓ cell apoptosis	[73]
Asiaticoside	*Centella asiatica* (L.) Urban	Ursane	Male SD rats (inject 60% acetic acid (0.12 mL/rat))		↓ MPO	[76]
			Male SD rats (inject 60% acetic acid (0.12 mL/rat))		↓ iNOS	[77]
Niga-ichigoside F1	Rubus plants	Ursane	Male SD rats (oral ethanol (4 mL/kg) and sodium salicylate (200 mg/kg)		↑ SOD, GSH-Px	[80]
			Male Swiss mice (oral EtOH/HCl (60%/0.3 M, 0.5 mL/rat))	98.45% ^2^ (30 mg/kg)		[81]
Lupeol	Cucumber, carrot, mango, strawberries, and olive	Lupane	Male Swiss albino mice (oral absolute ethanol (0.2 mL/mice))	69.3% ^2^ (30 mg/kg)	↑ NP-SH	[83]
Betulinic acid	*Betula platyphylla* Sukaczev	Lupane	Male Wistar albino rats (indomethacin (40 mg/kg))		↑ mucus, pH ↓ MDA	[86]
Friedelin	Celastraceae, Asteraceae, Fabaceae, and Myrtaceous plants	Friedelane	Wistar albino rats (oral 96% ethanol (5 mL/kg))	88.21% ^1^ (35 mg/kg)	↑ PGE2, NO, SOD, GSH-px, CAT, GSH, IL-10, mucus, pH ↓ MPO, MDA, TNF-α, IL-6, caspase-3	[88]
Azadiradione	*Azadirachta indica* A. Juss.	limonoids	SD rats (cold restraint)	58.5% ^1^ (40 mg/kg)	↑ pH, mucus, PGE2 ↓ H^+^/K^+^-ATPase	[90]
			SD rats (oral absolute ethanol (5 mL/kg))	71.67% ^1^ (20 mg/kg)		
			SD rats (pyloric ligation)	50.0% ^1^ (20 mg/kg)		
			SD rats (aspirin (150 mg/kg))	55.53% ^1^ (20 mg/kg)		

^1^ Reduction in ulcer index; ^2^ Reduction in ulcer area; ↓ indicates a decrease in expression and ↑ indicates an increase in expression.

## Abbreviations

The main abbreviations are listed in alphabetical order:

Bax	Bcl-2-associated X protein
Bcl-2	B-cell lymphoma-2
CAT	Catalase
COX-1	Cyclooxygenase-1
COX-2	Cyclooxygenase-2
EGF	Epidermal growth factor
EGFR	Epidermal growth factor receptor
ET-1	Endothelin-1
EtOH	Ethanol
Fas	Factor-related Apoptosis
FasL	Factor-related Apoptosis Ligand
GSH	Glutathione
GSH-Px	Glutathione peroxidase
GU	Gastric ulcer
*H. pylori*.	*Helicobacter pylori*
H_2_S	Hydrogen sulfide
HCl	Hydrochloric acid
HO-1	Heme oxygenase-1
HSP 70	Heat shock protein 70
IL-10	Interleukin-10
IL-1β	Interleukin-1β
IL-4	Interleukin-4
IL-6	Interleukin-6
iNOS	Inducible nitric oxide synthase
LTB4	Leukotriene B4
MCP-1	Monocyte chemoattractant protein-1
MDA	Malondialdehyde
MPO	Myeloperoxidase
NF-κB	Nuclear factor kappa-B
NO	Nitric oxide
NP-SH	Non-protein sulfhydryl groups
Nrf2	Nuclear factor erythroid 2-related factor 2
NSAIDs	Non-steroidal anti-inflammatory drugs
PGE2	Prostaglandin E2
PGs	Prostaglandins
ROS	Reactive Oxygen Species
SOD	Superoxide dismutase
TNF-α	Tumor necrosis factor α

## References

[1] Gong H., Zhao N., Zhu C., Luo L., Liu S. (2024). Treatment of gastric ulcer, traditional Chinese medicine may be a better choice. J. Ethnopharmacol..

[2] Hassan H.M., Alatawi N.M., Bagalagel A., Diri R., Noor A., Almasri D., Bakhsh H.T., Kutbi H.I., Ashy N., Al-Gayyar M.M.H. (2023). Genistein ameliorated experimentally induced gastric ulcer in rats via inhibiting gastric tissues fibrosis by modulating Wnt/β-catenin/TGF-β/PKB pathway. Redox Rep..

[3] Choudhary M.K., Bodakhe S.H., Gupta S.K. (2013). Assessment of the antiulcer potential of Moringa oleifera root-bark extract in rats. J. Acupunct. Meridian Stud..

[4] Zhou D., Yang Q., Tian T., Chang Y., Li Y., Duan L.R., Li H., Wang S.W. (2020). Gastroprotective effect of gallic acid against ethanol-induced gastric ulcer in rats: Involvement of the Nrf2/HO-1 signaling and anti-apoptosis role. Biomed. Pharmacother..

[5] Guo Y., Wu Y., Huang T., Huang D., Zeng Q., Wang Z., Hu Y., Liang P., Chen H., Zheng Z. (2024). Licorice flavonoid ameliorates ethanol-induced gastric ulcer in rats by suppressing apoptosis via PI3K/AKT signaling pathway. J. Ethnopharmacol..

[6] Feng L., A L., Li H., Mu X., Ta N., Bai L., Fu M., Chen Y. (2023). Pharmacological Mechanism of Aucklandiae Radix against Gastric Ulcer Based on Network Pharmacology and In Vivo Experiment. Medicina.

[7] Lanas A., Chan F.K.L. (2017). Peptic ulcer disease. Lancet.

[8] Liu Q., Tang J., Chen S., Hu S., Shen C., Xiang J., Chen N., Wang J., Ma X., Zhang Y. (2022). Berberine for gastric cancer prevention and treatment: Multi-step actions on the Correa’s cascade underlie its therapeutic effects. Pharmacol. Res..

[9] Paragomi P., Dabo B., Pelucchi C., Bonzi R., Bako A.T., Sanusi N.M., Nguyen Q.H., Zhang Z.F., Palli D., Ferraroni M. (2022). The Association between Peptic Ulcer Disease and Gastric Cancer: Results from the Stomach Cancer Pooling (StoP) Project Consortium. Cancers.

[10] Mousavi T., Nikfar S., Abdollahi M. (2022). The pharmacotherapeutic management of duodenal and gastric ulcers. Expert Opin. Pharmacother..

[11] Begg M., Tarhuni M., Fotso M.N., Gonzalez N.A., Sanivarapu R.R., Osman U., Latha Kumar A., Sadagopan A., Mahmoud A., Khan S. (2023). Comparing the Safety and Efficacy of Proton Pump Inhibitors and Histamine-2 Receptor Antagonists in the Management of Patients with Peptic Ulcer Disease: A Systematic Review. Cureus.

[12] Clarke K., Adler N., Agrawal D., Bhakta D., Sata S.S., Singh S., Gupta A., Pahwa A., Pherson E., Sun A. (2022). Indications for the Use of Proton Pump Inhibitors for Stress Ulcer Prophylaxis and Peptic Ulcer Bleeding in Hospitalized Patients. Am. J. Med..

[13] O’Connor A., O’Morain C.A., Ford A.C. (2017). Population screening and treatment of *Helicobacter pylori* infection. Nat. Rev. Gastroenterol. Hepatol..

[14] Luo J.H., Zou W.S., Li J., Liu W., Huang J., Wu H.W., Shen J.L., Li F., Yuan J.S., Tao A.K. (2023). Untargeted serum and liver metabolomics analyses reveal the gastroprotective effect of polysaccharide from Evodiae fructus on ethanol-induced gastric ulcer in mice. Int. J. Biol. Macromol..

[15] Yi L., Lu Y., Yu S., Cheng Q., Yi L. (2022). Formononetin inhibits inflammation and promotes gastric mucosal angiogenesis in gastric ulcer rats through regulating NF-κB signaling pathway. J. Recept. Signal Transduct. Res..

[16] Abd-Alla H.I., Ibrahim Fouad G., Ahmed K.A., Shaker K. (2022). Alloimperatorin from Ammi majus fruits mitigates Piroxicam-provoked gastric ulcer and hepatorenal toxicity in rats via suppressing oxidative stress and apoptosis. Biomarkers.

[17] Ren S., Wei Y., Niu M., Li R., Wang R., Wei S., Wen J., Wang D., Yang T., Chen X. (2021). Mechanism of rutaecarpine on ethanol-induced acute gastric ulcer using integrated metabolomics and network pharmacology. Biomed. Pharmacother..

[18] Szlasa W., Ślusarczyk S., Nawrot-Hadzik I., Abel R., Zalesińska A., Szewczyk A., Sauer N., Preissner R., Saczko J., Drąg M. (2023). Betulin and Its Derivatives Reduce Inflammation and COX-2 Activity in Macrophages. Inflammation.

[19] Li D., Guo Y.Y., Cen X.F., Qiu H.L., Chen S., Zeng X.F., Zeng Q., Xu M., Tang Q.Z. (2022). Lupeol protects against cardiac hypertrophy via TLR4-PI3K-Akt-NF-κB pathways. Acta Pharmacol. Sin..

[20] Zhou Q., Zhao Y., Fu X., Chen Q., Tang Y., Gao X. (2020). Naturally occurring triterpene Lupane exerts anticancer effects on colorectal cancer cells via induction of apoptosis and autophagy and suppresses cell migration and invasion by targeting MMP-9. J. BUON.

[21] Chung P.Y.K., Gan M.Y., Chin B.Y. (2022). Pentacyclic Triterpenoids as Antibiofilm Agents against Methicillinresistant and Biofilm-forming Staphylococcus aureus (MRSA). Curr. Pharm. Biotechnol..

[22] Abdullah, Khan M.A., Adhikari A. (2023). Radical Scavenging, Anti-Inflammatory, and Hepatoprotective Activities of Pentacyclic Triterpene isolated from Rosa webbiana. Curr. Drug Targets.

[23] Farina C., Pinza M., Pifferi G. (1998). Synthesis and anti-ulcer activity of new derivatives of glycyrrhetic, oleanolic and ursolic acids. Farmaco.

[24] Yao W., Guan Y. (2022). Ginsenosides in cancer: A focus on the regulation of cell metabolism. Biomed. Pharmacother..

[25] Shah M.A., Abuzar S.M., Ilyas K., Qadees I., Bilal M., Yousaf R., Kassim R.M.T., Rasul A., Saleem U., Alves M.S. (2023). Ginsenosides in cancer: Targeting cell cycle arrest and apoptosis. Chem. Biol. Interact..

[26] Jeong C.S., Hyun J.E., Kim Y.S. (2003). Ginsenoside Rb1: The anti-ulcer constituent from the head of Panax ginseng. Arch. Pharm. Res..

[27] Yoshikawa M., Sugimoto S., Nakamura S., Sakumae H., Matsuda H. (2007). Medicinal flowers. XVI. New dammarane-type triterpene tetraglycosides and gastroprotective principles from flower buds of Panax ginseng. Chem. Pharm. Bull..

[28] Zhang K., Liu Y., Wang C., Li J., Xiong L., Wang Z., Liu J., Li P. (2019). Evaluation of the gastroprotective effects of 20 (S)-ginsenoside Rg3 on gastric ulcer models in mice. J. Ginseng Res..

[29] Chen J., Duan Z., Liu Y., Fu R., Zhu C. (2022). Ginsenoside Rh4 Suppresses Metastasis of Esophageal Cancer and Expression of c-Myc via Targeting the Wnt/β-Catenin Signaling Pathway. Nutrients.

[30] Dong F., Qu L., Duan Z., He Y., Ma X., Fan D. (2023). Ginsenoside Rh4 inhibits breast cancer growth through targeting histone deacetylase 2 to regulate immune microenvironment and apoptosis. Bioorg. Chem..

[31] Wu Y., Duan Z., Qu L., Zhang Y., Zhu C., Fan D. (2023). Gastroprotective effects of ginsenoside Rh4 against ethanol-induced gastric mucosal injury by inhibiting the MAPK/NF-κB signaling pathway. Food Funct..

[32] Gao Y., Chu S.F., Li J.P., Zhang Z., Yan J.Q., Wen Z.L., Xia C.Y., Mou Z., Wang Z.Z., He W.B. (2015). Protopanaxtriol protects against 3-nitropropionic acid-induced oxidative stress in a rat model of Huntington’s disease. Acta Pharmacol. Sin..

[33] Lu B., Wang D., Xie D., Wu C., Sun M. (2023). 20(S)-Protopanaxatriol ameliorates MAFLD by inhibiting NLRP3 inflammasome. Eur. J. Pharmacol..

[34] Wang C., Tan L., Liu J., Fu D., Wang C., Li P., Li Z., Liu J. (2022). Integrated Metabolomics and Network Pharmacology to Decipher the Latent Mechanisms of Protopanaxatriol against Acetic Acid-Induced Gastric Ulcer. Int. J. Mol. Sci..

[35] Liu J., Xu Y., Yang J., Wang W., Zhang J., Zhang R., Meng Q. (2017). Discovery, semisynthesis, biological activities, and metabolism of ocotillol-type saponins. J. Ginseng Res..

[36] Cao Y., Wang K., Xu S., Kong L., Bi Y., Li X. (2020). Recent Advances in the Semisynthesis, Modifications and Biological Activities of Ocotillol-Type Triterpenoids. Molecules.

[37] Wang C., Yuan Y., Pan H., Hsu A.C., Chen J., Liu J., Li P., Wang F. (2020). Protective Effect of Ocotillol, the Derivate of Ocotillol-Type Saponins in Panax Genus, against Acetic Acid-Induced Gastric Ulcer in Rats Based on Untargeted Metabolomics. Int. J. Mol. Sci..

[38] Zhang J., Wu C., Gao L., Du G., Qin X. (2020). Astragaloside IV derived from Astragalus membranaceus: A research review on the pharmacological effects. Adv. Pharmacol..

[39] Liang Y., Chen B., Liang D., Quan X., Gu R., Meng Z., Gan H., Wu Z., Sun Y., Liu S. (2023). Pharmacological Effects of Astragaloside IV: A Review. Molecules.

[40] Navarrete A., Arrieta J., Terrones L., Abou-Gazar H., Calis I. (2005). Gastroprotective effect of Astragaloside IV: Role of prostaglandins, sulfhydryls and nitric oxide. J. Pharm. Pharmacol..

[41] Mao S., Yang G., Li W., Zhang J., Liang H., Li J., Zhang M. (2016). Gastroprotective Effects of Astragaloside IV against Acute Gastric Lesion in Rats. PLoS ONE.

[42] Fan D.D., Lin S., Song Y.P., Wang Z.Y., Liu B., Gao S.N., Fan Y.H., Zhu S., Li S., Jiang L. (2016). Astragaloside IV protects rat gastric mucosa against aspirin-induced damage. Int. Immunopharmacol..

[43] Navarrete A., Martínez-Uribe L., Reyes-Trejo B. (1998). Gastroprotective Activity of the Stem Bark of *Amphipterygium adstringens* in Rats. Phytoteraphy Res..

[44] Arrieta J., Benitez J., Flores E., Castillo C., Navarrete A. (2003). Purification of gastroprotective triterpenoids from the stem bark of *Amphipterygium adstringens*; role of prostaglandins, sulfhydryls, nitric oxide and capsaicin-sensitive neurons. Planta Med..

[45] Pineda-Peña E.A., Orona-Ortiz A., Velázquez-Moyado J.A., Tavares-Carvalho J.C., Chávez-Piña A.E., Balderas-López J.L., Navarrete A. (2020). Anti-inflammatory, antioxidant, and gaso-protective mechanism of 3α-hydroxymasticadienoic acid and diligustilide combination on indomethacin gastric damage. Naunyn Schmiedebergs Arch. Pharmacol..

[46] Pollier J., Goossens A. (2012). Oleanolic acid. Phytochemistry.

[47] Castellano J.M., Ramos-Romero S., Perona J.S. (2022). Oleanolic Acid: Extraction, Characterization and Biological Activity. Nutrients.

[48] Astudillo L., Rodriguez J.A., Schmeda-Hirschmann G. (2002). Gastroprotective activity of oleanolic acid derivatives on experimentally induced gastric lesions in rats and mice. J. Pharm. Pharmacol..

[49] Rodríguez J.A., Astudillo L., Schmeda-Hirschmann G. (2003). Oleanolic acid promotes healing of acetic acid-induced chronic gastric lesions in rats. Pharmacol. Res..

[50] Sánchez M., Theoduloz C., Schmeda-Hirschmann G., Razmilic I., Yáñez T., Rodríguez J.A. (2006). Gastroprotective and ulcer-healing activity of oleanolic acid derivatives: In Vitro-In Vivo relationships. Life Sci..

[51] Tian Y., Zhang X., Du M., Li F., Xiao M., Zhang W. (2021). Synergistic Antioxidant Effects of Araloside A and L-Ascorbic Acid on H(2)O(2)-Induced HEK293 Cells: Regulation of Cellular Antioxidant Status. Oxid. Med. Cell. Longev..

[52] Lee E.B., Kim O.J., Kang S.S., Jeong C. (2005). Araloside A, an antiulcer constituent from the root bark of Aralia elata. Biol. Pharm. Bull..

[53] He H., Li X., Yu H., Zhu S., He Y., Komatsu K., Guo D., Li X., Wang J., Luo H. (2019). Gastroprotective effect of araloside A on ethanol- and aspirin-induced gastric ulcer in mice: Involvement of H(+)/K(+)-ATPase and mitochondrial-mediated signaling pathway. J. Nat. Med..

[54] Ding Y., Brand E., Wang W., Zhao Z. (2022). Licorice: Resources, applications in ancient and modern times. J. Ethnopharmacol..

[55] Chen L., Gong J., Yong X., Li Y., Wang S. (2024). A review of typical biological activities of glycyrrhetinic acid and its derivatives. RSC Adv..

[56] Yano S., Harada M., Watanabe K., Nakamaru K., Hatakeyama Y., Shibata S., Takahashi K., Mori T., Hirabayashi K., Takeda M. (1989). Antiulcer activities of glycyrrhetinic acid derivatives in experimental gastric lesion models. Chem. Pharm. Bull..

[57] Dehpour A.R., Zolfaghari M.E., Samadian T., Kobarfard F., Faizi M., Assari M. (1995). Antiulcer activities of liquorice and its derivatives in experimental gastric lesion induced by ibuprofen in rats. Int. J. Pharm..

[58] Krausse R., Bielenberg J., Blaschek W., Ullmann U. (2004). In vitro anti-*Helicobacter pylori* activity of Extractum liquiritiae, glycyrrhizin and its metabolites. J. Antimicrob. Chemother..

[59] Cao D., Jiang J., You L., Jia Z., Tsukamoto T., Cai H., Wang S., Hou Z., Suo Y.E., Cao X. (2016). The Protective Effects of 18β-Glycyrrhetinic Acid on *Helicobacter pylori*-Infected Gastric Mucosa in Mongolian Gerbils. Biomed Res. Int..

[60] MacDonell E.C., Rajcan I. (2018). Identification of quantitative trait loci associated with soyasaponin I concentration in soybean seed. Theor. Appl. Genet..

[61] Morsi A.A., Shawky L.M., Shawky T.M., Bahr M.H., Alnasr M.T.A., El Bana E. (2024). Targeting NF-κB/COX-2 signaling by soyasaponin I alleviates diclofenac-induced gastric ulceration in male albino rats. Cell Biochem. Funct..

[62] Niu X., Yao H., Li W., Mu Q., Li H., Hu H., Li Y., Huang H. (2014). δ-Amyrone, a specific inhibitor of cyclooxygenase-2, exhibits anti-inflammatory effects in vitro and in vivo of mice. Int. Immunopharmacol..

[63] Li W., Yao H., Niu X., Wang Y., Zhang H., Li H., Mu Q. (2015). Protective effect of δ-amyrone against ethanol-induced gastric ulcer in mice. Immunobiology.

[64] Lozano-Mena G., Sánchez-González M., Juan M.E., Planas J.M. (2014). Maslinic acid, a natural phytoalexin-type triterpene from olives—A promising nutraceutical?. Molecules.

[65] da Rosa R.L., Nesello LÂ N., Mariano L.N.B., Somensi L.B., Campos A., Pinheiro A.M., Costa S., Rial M., Tozzo M., Cechinel-Filho V. (2018). Gastroprotective activity of the methanol extract from peels of Plinia edulis (Vell.) Sobral fruits and its isolated triterpenes: Maslinic and ursolic acids. Naunyn Schmiedebergs Arch. Pharmacol..

[66] Kosolapov D., Jáč P., Riasová P., Poušková J., Polášek M., Nováková L. (2024). Advances and Challenges in the Analysis of Boswellic Acids by Separation Methods. Crit. Rev. Anal. Chem..

[67] Zhang Y., Jia J., Ding Y., Ma Y., Shang P., Liu T., Hui G., Wang L., Wang M., Zhu Z. (2016). Alpha-boswellic acid protects against ethanol-induced gastric injury in rats: Involvement of nuclear factor erythroid-2-related factor 2/heme oxygenase-1 pathway. J. Pharm. Pharmacol..

[68] Seo D.Y., Lee S.R., Heo J.W., No M.H., Rhee B.D., Ko K.S., Kwak H.B., Han J. (2018). Ursolic acid in health and disease. Korean J. Physiol. Pharmacol..

[69] Kashyap D., Tuli H.S., Sharma A.K. (2016). Ursolic acid (UA): A metabolite with promising therapeutic potential. Life Sci..

[70] Pandey D., Joshi A., Hemalatha S. (2017). Anti-Ulcer Study of Standardized Ethanol Root Extract of Aganosma Dichotoma and Isolated Ursolic Acid. Int. J. Pharm. Pharm. Sci..

[71] Elshamy A.I., Farrag A.-R.H., Mohamed S.H., Ali N.A., Mohamed T.A., Menshawy M.M., Zaglool A.W., Efferth T., Hegazy M.-E.F. (2020). Gastroprotective effects of ursolic acid isolated from Ochrosia elliptica on ethanol-induced gastric ulcer in rats. Med. Chem. Res..

[72] Olech M., Ziemichód W., Nowacka-Jechalke N. (2021). The Occurrence and Biological Activity of Tormentic Acid-A Review. Molecules.

[73] He J.Y., Li J., Zhang Y.Y., He H.B., He Y.M., Xu D.X., Wang X., Wu H.Y., Zhang J.H., Jahid H. (2023). Tormentic acid, a triterpenoid isolated from the fruits of Chaenomeles speciose, protected indomethacin-induced gastric mucosal lesion via modulating miR-139 and the CXCR4/CXCL12/PLC/PKC/Rho a/MLC pathway. Pharm. Biol..

[74] Sun B., Wu L., Wu Y., Zhang C., Qin L., Hayashi M., Kudo M., Gao M., Liu T. (2020). Therapeutic Potential of *Centella asiatica* and Its Triterpenes: A Review. Front. Pharmacol..

[75] He Z., Hu Y., Niu Z., Zhong K., Liu T., Yang M., Ji L., Hu W. (2023). A review of pharmacokinetic and pharmacological properties of asiaticoside, a major active constituent of *Centella asiatica* (L.) Urb. J. Ethnopharmacol..

[76] Cheng C.L., Guo J.S., Luk J., Koo M.W. (2004). The healing effects of Centella extract and asiaticoside on acetic acid induced gastric ulcers in rats. Life Sci..

[77] Guo J.S., Cheng C.L., Koo M.W. (2004). Inhibitory effects of *Centella asiatica* water extract and asiaticoside on inducible nitric oxide synthase during gastric ulcer healing in rats. Planta Med..

[78] Tonin T.D., Thiesen L.C., de Oliveira Nunes M.L., Broering M.F., Donato M.P., Goss M.J., Petreanu M., Niero R., Machado I.D., Santin J.R. (2016). Rubus imperialis (Rosaceae) extract and pure compound niga-ichigoside F1: Wound healing and anti-inflammatory effects. Naunyn Schmiedebergs Arch. Pharmacol..

[79] Xia S.F., Shao J., Zhao S.Y., Qiu Y.Y., Teng L.P., Huang W., Wang S.S., Cheng X.R., Jiang Y.Y. (2018). Niga-ichigoside F1 ameliorates high-fat diet-induced hepatic steatosis in male mice by Nrf2 activation. Food Funct..

[80] Nam J.H., Jung H.J., Choi J., Lee K.T., Park H.J. (2006). The anti-gastropathic and anti-rheumatic effect of niga-ichigoside F1 and 23-hydroxytormentic acid isolated from the unripe fruits of Rubus coreanus in a rat model. Biol. Pharm. Bull..

[81] Berté P.E., da Silva Lopes J., Comandulli N.G., Rangel D.W., Monache F.D., Filho V.C., Niero R., de Andrade S.F. (2014). Evaluation of the gastroprotective activity of the extracts, fractions, and pure compounds obtained from aerial parts of Rubus imperialis in different experimental models. Naunyn Schmiedebergs Arch. Pharmacol..

[82] Sohag A.A.M., Hossain M.T., Rahaman M.A., Rahman P., Hasan M.S., Das R.C., Khan M.K., Sikder M.H., Alam M., Uddin M.J. (2022). Molecular pharmacology and therapeutic advances of the pentacyclic triterpene lupeol. Phytomedicine.

[83] Lira S.R., Rao V.S., Carvalho A.C., Guedes M.M., de Morais T.C., de Souza A.L., Trevisan M.T., Lima A.F., Chaves M.H., Santos F.A. (2009). Gastroprotective effect of lupeol on ethanol-induced gastric damage and the underlying mechanism. Inflammopharmacology.

[84] Oluwasegun A., Ogochukwu U., Ugochukwu O., Mussaddiq I., Bunyamin A. (2023). Lupeol: A Triterpenoid Isolated from the Stem Bark of Hymenocardia Acida (tul.) Exhibits a van der Waal Antagonism on the Alpha Subunit of Gastric H+K+Atpase—A Promising Antiulcer Principle. Drug Res..

[85] Lou H., Li H., Zhang S., Lu H., Chen Q. (2021). A Review on Preparation of Betulinic Acid and Its Biological Activities. Molecules.

[86] Onwuchekwa C., Oluwole F.S. (2015). Anti-Gastric Ulcer Effect of Betulinic Acid in Male Albino Rats. Niger. J. Physiol. Sci..

[87] Singh S.K., Shrivastava S., Mishra A.K., Kumar D., Pandey V.K., Srivastava P., Pradhan B., Behera B.C., Bahuguna A., Baek K.H. (2023). Friedelin: Structure, Biosynthesis, Extraction, and Its Potential Health Impact. Molecules.

[88] Antonisamy P., Duraipandiyan V., Aravinthan A., Al-Dhabi N.A., Ignacimuthu S., Choi K.C., Kim J.H. (2015). Protective effects of friedelin isolated from Azima tetracantha Lam. against ethanol-induced gastric ulcer in rats and possible underlying mechanisms. Eur. J. Pharmacol..

[89] Lin M., Yang S., Huang J., Zhou L. (2021). Insecticidal Triterpenes in Meliaceae: Plant Species, Molecules and Activities: Part I (Aphanamixis-Chukrasia). Int. J. Mol. Sci..

[90] Singh R., Mishra V., Pandeti S., Palit G., Barthwal M.K., Pandey H.P., Narender T. (2015). Cytoprotective and Anti-secretory Effects of Azadiradione Isolated from the Seeds of *Azadirachta indica* (neem) on Gastric Ulcers in Rat Models. Phytother. Res..

[91] Yang S., Lian G. (2020). ROS and diseases: Role in metabolism and energy supply. Mol. Cell. Biochem..

[92] Ermis A., Aritici Colak G., Acikel-Elmas M., Arbak S., Kolgazi M. (2023). Ferulic Acid Treats Gastric Ulcer via Suppressing Oxidative Stress and Inflammation. Life.

[93] Ndrepepa G. (2019). Myeloperoxidase—A bridge linking inflammation and oxidative stress with cardiovascular disease. Clin. Chim. Acta.

[94] Liu T., Sun L., Zhang Y., Wang Y., Zheng J. (2022). Imbalanced GSH/ROS and sequential cell death. J. Biochem. Mol. Toxicol..

[95] Zheng Y.F., Xie J.H., Xu Y.F., Liang Y.Z., Mo Z.Z., Jiang W.W., Chen X.Y., Liu Y.H., Yu X.D., Huang P. (2014). Gastroprotective effect and mechanism of patchouli alcohol against ethanol, indomethacin and stress-induced ulcer in rats. Chem. Biol. Interact..

[96] Balan T., Mohd Sani M.H., Suppaiah V., Mohtarrudin N., Suhaili Z., Ahmad Z., Zakaria Z.A. (2013). Antiulcer activity of Muntingia calabura leaves involves the modulation of endogenous nitric oxide and nonprotein sulfhydryl compounds. Pharm. Biol..

[97] Hobani Y.H., Mohan S., Shaheen E., Abdelhaleem A., Faruque Ahmad M., Bhatia S., Abou-Elhamd A.S. (2022). Gastroprotective effect of low dose Eugenol in experimental rats against ethanol induced toxicity: Involvement of antiinflammatory and antioxidant mechanism. J. Ethnopharmacol..

[98] Yanaka A. (2018). Role of NRF2 in protection of the gastrointestinal tract against oxidative stress. J. Clin. Biochem. Nutr..

[99] Ye H.Y., Shang Z.Z., Zhang F.Y., Zha X.Q., Li Q.M., Luo J.P. (2023). Dendrobium huoshanense stem polysaccharide ameliorates alcohol-induced gastric ulcer in rats through Nrf2-mediated strengthening of gastric mucosal barrier. Int. J. Biol. Macromol..

[100] Sallam A.M., Darwish S.F., El-Dakroury W.A., Radwan E. (2021). Olmesartan niosomes ameliorates the Indomethacin-induced gastric ulcer in rats: Insights on MAPK and Nrf2/HO-1 signaling pathway. Pharm. Res..

[101] Yang T., Wang R., Liu H., Wang L., Li J., Wu S., Chen X., Yang X., Zhao Y. (2021). Berberine regulates macrophage polarization through IL-4-STAT6 signaling pathway in *Helicobacter pylori*-induced chronic atrophic gastritis. Life Sci..

[102] Bosco M.C. (2019). Macrophage polarization: Reaching across the aisle?. J. Allergy Clin. Immunol..

[103] Wang C., Peng D., Liu Y., Wu Y., Guo P., Wei J. (2021). Agarwood Alcohol Extract Protects against Gastric Ulcer by Inhibiting Oxidation and Inflammation. Evid. Based Complement. Alternat. Med..

[104] Singh S., Anshita D., Ravichandiran V. (2021). MCP-1: Function, regulation, and involvement in disease. Int. Immunopharmacol..

[105] Watanabe T., Higuchi K., Hamaguchi M., Shiba M., Tominaga K., Fujiwara Y., Matsumoto T., Arakawa T. (2004). Monocyte chemotactic protein-1 regulates leukocyte recruitment during gastric ulcer recurrence induced by tumor necrosis factor-alpha. Am. J. Physiol. Gastrointest. Liver Physiol..

[106] Wan M., Tang X., Stsiapanava A., Haeggström J.Z. (2017). Biosynthesis of leukotriene B(4). Semin. Immunol..

[107] Pineda-Peña E.A., Martínez-Pérez Y., Galicia-Moreno M., Navarrete A., Segovia J., Muriel P., Favari L., Castañeda-Hernández G., Chávez-Piña A.E. (2018). Participation of the anti-inflammatory and antioxidative activity of docosahexaenoic acid on indomethacin-induced gastric injury model. Eur. J. Pharmacol..

[108] Wang W., Wang S.K., Wang Q., Zhang Z., Li B., Zhou Z.D., Zhang J.F., Lin C., Chen T.X., Jin Z. (2023). Diclofenac and eugenol hybrid with enhanced anti-inflammatory activity through activating HO-1 and inhibiting NF-κB pathway in vitro and in vivo. Eur. J. Med. Chem..

[109] Minatel I.O., Francisqueti F.V., Corrêa C.R., Lima G.P. (2016). Antioxidant Activity of γ-Oryzanol: A Complex Network of Interactions. Int. J. Mol. Sci..

[110] Ren S., Wei Y., Wang R., Wei S., Wen J., Yang T., Chen X., Wu S., Jing M., Li H. (2020). Rutaecarpine Ameliorates Ethanol-Induced Gastric Mucosal Injury in Mice by Modulating Genes Related to Inflammation, Oxidative Stress and Apoptosis. Front. Pharmacol..

[111] Yu L., Li R., Liu W., Zhou Y., Li Y., Qin Y., Chen Y., Xu Y. (2020). Protective Effects of Wheat Peptides against Ethanol-Induced Gastric Mucosal Lesions in Rats: Vasodilation and Anti-Inflammation. Nutrients.

[112] Du Y., Zhao W., Lu L., Zheng J., Hu X., Yu Z., Zhu L. (2013). Study on the antiulcer effects of Veronicastrum axillare on gastric ulcer in rats induced by ethanol based on tumor necrosis factor-α (TNF-α) and endothelin-1 (ET-1). Asian Pac. J. Trop Biomed..

[113] Nishida T., Tsuji S., Kimura A., Tsujii M., Ishii S., Yoshio T., Shinzaki S., Egawa S., Irie T., Yasumaru M. (2006). Endothelin-1, an ulcer inducer, promotes gastric ulcer healing via mobilizing gastric myofibroblasts and stimulates production of stroma-derived factors. Am. J. Physiol. Gastrointest. Liver Physiol..

[114] Banecki K., Dora K.A. (2023). Endothelin-1 in Health and Disease. Int. J. Mol. Sci..

[115] Isik M., Ozbayer C., Donmez D.B., Colak E., Ustuner M.C., Erol K., Degirmenci I. (2022). Effects of the probiotic, Lactobacillus rhamnosus GG, on ulcer pathogenesis, HSP70 stress protein and nitric oxide levels in stress induced ulcer. Biotech. Histochem..

[116] Abo-Elsoud R., Ahmed Mohamed Abdelaziz S., Attia Abd Eldaim M., Hazzaa S.M. (2022). Moringa oleifera alcoholic extract protected stomach from bisphenol A-induced gastric ulcer in rats via its anti-oxidant and anti-inflammatory activities. Environ. Sci. Pollut. Res. Int..

[117] Chen G., Xie X., Peng F., Wang T., Chen J., Li G., Liu J., Peng C. (2022). Protective effect of the combination of essential oil from patchouli and tangerine peel against gastric ulcer in rats. J. Ethnopharmacol..

[118] Mabrok H.B., Mohamed M.S. (2019). Induction of COX-1, suppression of COX-2 and pro-inflammatory cytokines gene expression by moringa leaves and its aqueous extract in aspirin-induced gastric ulcer rats. Mol. Biol. Rep..

[119] Suleyman H., Albayrak A., Bilici M., Cadirci E., Halici Z. (2010). Different mechanisms in formation and prevention of indomethacin-induced gastric ulcers. Inflammation.

[120] Takeuchi K., Amagase K. (2018). Roles of Cyclooxygenase, Prostaglandin E2 and EP Receptors in Mucosal Protection and Ulcer Healing in the Gastrointestinal Tract. Curr. Pharm. Des..

[121] Dejban P., Eslami F., Rahimi N., Takzare N., Jahansouz M., Dehpour A.R. (2020). Involvement of nitric oxide pathway in the anti-inflammatory effect of modafinil on indomethacin-, stress-, and ethanol-induced gastric mucosal injury in rat. Eur. J. Pharmacol..

[122] Liang T.Y., Deng R.M., Li X., Xu X., Chen G. (2021). The role of nitric oxide in peptic ulcer: A narrative review. Med. Gas Res..

[123] Lee J.M., Lim J.Y., Kim Y., Kim Y.J., Choi H.S., Kim E.S., Keum B., Seo Y.S., Jeen Y.T., Lee H.S. (2016). Benexate hydrochloride betadex modulates nitric oxide synthesis and cytokine expression in gastric ulcers. Exp. Ther. Med..

[124] Lima-Júnior R.C., Figueiredo A.A., Freitas H.C., Melo M.L., Wong D.V., Leite C.A., Medeiros R.P., Marques-Neto R.D., Vale M.L., Brito G.A. (2012). Involvement of nitric oxide on the pathogenesis of irinotecan-induced intestinal mucositis: Role of cytokines on inducible nitric oxide synthase activation. Cancer Chemother. Pharmacol..

[125] Magierowski M., Magierowska K., Kwiecien S., Brzozowski T. (2015). Gaseous mediators nitric oxide and hydrogen sulfide in the mechanism of gastrointestinal integrity, protection and ulcer healing. Molecules.

[126] Jabbar A.A.J., Mothana R.A., Ameen Abdulla M., Othman Abdullah F., Abdul-Aziz Ahmed K., Rizgar Hussen R., Hawwal M.F., Fantoukh O.I., Hasson S. (2023). Mechanisms of anti-ulcer actions of *Prangos pabularia* (L.) in ethanol-induced gastric ulcer in rats. Saudi Pharm. J..

[127] Abdoulrahman K. (2023). Anti-ulcer effect of Ranunculus millefoliatus on absolute alcohol-induced stomach ulceration. Saudi J. Biol. Sci..

[128] El-Shiekh R.A., Salama A., Al-Mokaddem A.K., Bader A., Abdel-Sattar E.A., Russelioside B. (2021). A pregnane glycoside for treatment of gastric ulcer via modulation of heat shock protein-70 and vascular endothelial growth factor. Steroids.

[129] Ishihara T., Suemasu S., Asano T., Tanaka K., Mizushima T. (2011). Stimulation of gastric ulcer healing by heat shock protein 70. Biochem. Pharmacol..

[130] Herszényi L., Bakucz T., Barabás L., Tulassay Z. (2020). Pharmacological Approach to Gastric Acid Suppression: Past, Present, and Future. Dig. Dis..

[131] Engevik A.C., Kaji I., Goldenring J.R. (2020). The Physiology of the Gastric Parietal Cell. Physiol. Rev..

[132] Mahmoud M.F., Abdo W., Nabil M., Drissi B., El-Shazly A.M., Abdelfattah M.A.O., Sobeh M. (2023). Apple (*Malus domestica* Borkh) leaves attenuate indomethacin-induced gastric ulcer in rats. Biomed. Pharmacother..

[133] Pelaseyed T., Bergström J.H., Gustafsson J.K., Ermund A., Birchenough G.M., Schütte A., van der Post S., Svensson F., Rodríguez-Piñeiro A.M., Nyström E.E. (2014). The mucus and mucins of the goblet cells and enterocytes provide the first defense line of the gastrointestinal tract and interact with the immune system. Immunol. Rev..

[134] Somensi L.B., Costa P., Boeing T., Bolda Mariano L.N., de Gregório E., ATM E.S., Longo B., Locatelli C., de Souza P., Magalhães C.G. (2022). Lupeol Stearate Accelerates Healing and Prevents Recurrence of Gastric Ulcer in Rodents. Evid. Based Complement. Alternat. Med..

[135] Tang S., Hu J., Meng Q., Dong X., Wang K., Qi Y., Chu C., Zhang X., Hou L. (2013). Daidzein induced apoptosis via down-regulation of Bcl-2/Bax and triggering of the mitochondrial pathway in BGC-823 cells. Cell Biochem. Biophys..

[136] Ma N., Sun Y., Yi J., Zhou L., Cai S. (2022). Chinese sumac (*Rhus chinensis* Mill.) fruits alleviate indomethacin-induced gastric ulcer in mice by improving oxidative stress, inflammation and apoptosis. J. Ethnopharmacol..

[137] Sahoo G., Samal D., Khandayataray P., Murthy M.K. (2023). A Review on Caspases: Key Regulators of Biological Activities and Apoptosis. Mol. Neurobiol..

[138] Luo Y., Fu X., Ru R., Han B., Zhang F., Yuan L., Men H., Zhang S., Tian S., Dong B. (2020). CpG Oligodeoxynucleotides Induces Apoptosis of Human Bladder Cancer Cells via Caspase-3-Bax/Bcl-2-p53 Axis. Arch. Med. Res..

[139] Cho J., Prashar A., Jones N.L., Moss S.F. (2021). *Helicobacter pylori* Infection. Gastroenterol. Clin. N. Am..

[140] Huang Y., Wang Q.L., Cheng D.D., Xu W.T., Lu N.H. (2016). Adhesion and Invasion of Gastric Mucosa Epithelial Cells by *Helicobacter pylori*. Front. Cell. Infect. Microbiol..

[141] Ansari S., Yamaoka Y. (2019). *Helicobacter pylori* Virulence Factors Exploiting Gastric Colonization and its Pathogenicity. Toxins.

[142] da Costa D.M., Pereira Edos S., Rabenhorst S.H. (2015). What exists beyond cagA and vacA?. Helicobacter pylori genes in gastric diseases. World J. Gastroenterol..

[143] Wu Y., Guo Y., Huang T., Huang D., Liu L., Shen C., Jiang C., Wang Z., Chen H., Liang P. (2023). Licorice flavonoid alleviates gastric ulcers by producing changes in gut microbiota and promoting mucus cell regeneration. Biomed. Pharmacother..

[144] Tarnawski A.S., Ahluwalia A. (2021). The Critical Role of Growth Factors in Gastric Ulcer Healing: The Cellular and Molecular Mechanisms and Potential Clinical Implications. Cells.

[145] Selim H.M., Negm W.A., Hawwal M.F., Hussein I.A., Elekhnawy E., Ulber R., Zayed A. (2023). Fucoidan mitigates gastric ulcer injury through managing inflammation, oxidative stress, and NLRP3-mediated pyroptosis. Int. Immunopharmacol..

[146] Liu J., Guo M., Fan X. (2021). Ethanol induces necroptosis in gastric epithelial cells in vitro. J. Food Biochem..

[147] Shams S.G.E., Eissa R.G. (2022). Amelioration of ethanol-induced gastric ulcer in rats by quercetin: Implication of Nrf2/HO1 and HMGB1/TLR4/NF-κB pathways. Heliyon.

[148] Tamura M., Ito H., Matsui H., Hyodo I. (2014). Acetaldehyde is an oxidative stressor for gastric epithelial cells. J. Clin. Biochem. Nutr..

[149] Zhang Y., Yuan Z., Chai J., Zhu D., Miao X., Zhou J., Gu X. (2023). ALDH2 ameliorates ethanol-induced gastric ulcer through suppressing NLPR3 inflammasome activation and ferroptosis. Arch. Biochem. Biophys..

[150] Bindu S., Mazumder S., Bandyopadhyay U. (2020). Non-steroidal anti-inflammatory drugs (NSAIDs) and organ damage: A current perspective. Biochem. Pharmacol..

[151] Mahmoud Y.I., Abd El-Ghffar E.A. (2019). Spirulina ameliorates aspirin-induced gastric ulcer in albino mice by alleviating oxidative stress and inflammation. Biomed. Pharmacother..

[152] Ruiz-Hurtado P.A., Garduño-Siciliano L., Domínguez-Verano P., Balderas-Cordero D., Gorgua-Jiménez G., Canales-Álvarez O., Canales-Martínez M.M., Rodríguez-Monroy M.A. (2021). Propolis and Its Gastroprotective Effects on NSAID-Induced Gastric Ulcer Disease: A Systematic Review. Nutrients.

[153] Musumba C., Pritchard D.M., Pirmohamed M. (2009). Review article: Cellular and molecular mechanisms of NSAID-induced peptic ulcers. Aliment. Pharmacol. Ther..

[154] Takeuchi K. (2012). Pathogenesis of NSAID-induced gastric damage: Importance of cyclooxygenase inhibition and gastric hypermotility. World J. Gastroenterol..

[155] Al-Gabri N., Elnagar G.M., Saghir S.A.M., El-Shaibany A., Alnomasy S.F., Althafar Z.M., Elkomy N., Elaasser M.M., Abdoh M.S., Yosri M. (2022). Preliminary Study of Gastroprotective Effect of Aloe perryi and Date Palm Extracts on Pyloric Ligation-Induced Gastric Ulcer in Experimental Rats. Biomed Res. Int..

[156] Asaad G.F., Saleh D.O., Mostafa R.E., Hassan A., Jaleel G.A. (2024). Pylorus ligation-induced hyperacidity: Synergistic prophylactic effects of linagliptin and L-arginine via up-regulation of EP4 receptor subtype and improvement of vascular endothelial damage. Naunyn Schmiedebergs Arch. Pharmacol..

[157] De Sales I.R.P., Formiga R.O., Machado F.D.F., Nascimento R.F., Pessoa M.M.B., Barros M., Vieira G.C., Gadelha F., Marinho A.F., Barbosa Filho J.M. (2018). Cytoprotective, antioxidant and anti-inflammatory mechanism related to antiulcer activity of *Cissampelos sympodialis* Eichl. in animal models. J. Ethnopharmacol..

[158] Wang X.Y., Yin J.Y., Zhao M.M., Liu S.Y., Nie S.P., Xie M.Y. (2018). Gastroprotective activity of polysaccharide from *Hericium erinaceus* against ethanol-induced gastric mucosal lesion and pylorus ligation-induced gastric ulcer, and its antioxidant activities. Carbohydr. Polym..

[159] Zaghlool S., Shehata B., Abo-Seif A., Abd El-Latif H. (2015). Comparison between the Protective Effects of Famotidine, Ginger and Marshmallow on Pyloric Ligation-Induced Peptic Ulcer in Rats. J. Bioequiv. Availab..

[160] Lu Q., Tang H. (2024). Overexpression of HSP27 accelerates stress-induced gastric ulcer healing via the CXCL12/CXCR4 axis. Clin. Exp. Pharmacol. Physiol..

[161] Jia Y.T., Ma B., Wei W., Xu Y., Wang Y., Tang H.T., Xia Z.F. (2007). Sustained activation of nuclear factor-kappaB by reactive oxygen species is involved in the pathogenesis of stress-induced gastric damage in rats. Crit. Care Med..

[162] Zhao D.Q., Xue H., Sun H.J. (2020). Nervous mechanisms of restraint water-immersion stress-induced gastric mucosal lesion. World J. Gastroenterol..

[163] Akmal M.N., Abdel Aziz I., Nur Azlina M.F. (2022). Piper sarmentosum Roxb. methanolic extract prevents stress-induced gastric ulcer by modulating oxidative stress and inflammation. Front. Pharmacol..

[164] Yu Y., Jia T.Z., Cai Q., Jiang N., Ma M.Y., Min D.Y., Yuan Y. (2015). Comparison of the anti-ulcer activity between the crude and bran-processed Atractylodes lancea in the rat model of gastric ulcer induced by acetic acid. J. Ethnopharmacol..

[165] Wang X.Y., Wang M., Yin J.Y., Song Y.H., Wang Y.X., Nie S.P., Xie M.Y. (2022). Gastroprotective activity of polysaccharide from the fruiting body of *Hericium erinaceus* against acetic acid-induced gastric ulcer in rats and structure of one bioactive fraction. Int. J. Biol. Macromol..

[166] Xue Z., Shi G., Fang Y., Liu X., Zhou X., Feng S., Zhao L. (2019). Protective effect of polysaccharides from Radix Hedysari on gastric ulcers induced by acetic acid in rats. Food Funct..

[167] Kowalska A., Kalinowska-Lis U. (2019). 18β-Glycyrrhetinic acid: Its core biological properties and dermatological applications. Int. J. Cosmet. Sci..

[168] Mancuso C., Santangelo R. (2017). Panax ginseng and Panax quinquefolius: From pharmacology to toxicology. Food Chem. Toxicol..

[169] Cao Y., Tao F., Yu Y., Song L., Zhang R., Feng J., Zhai Q., Xue P. (2023). Safety evaluation of rare ginsenosides of stems and leaves from American ginseng: 90-day exposure toxicity study combined with intestinal flora analysis and metabonomics in rats. Ecotoxicol. Environ. Saf..

[170] Zhang S., Zhao Y. (2016). Drug Metabolism and Pharmacokinetics of Dammarane Triterpenoids. Curr. Drug Metab..

[171] Jing L., Shuang G., Jing-bo L. (2009). Acute and Genetic Toxicity of Ursolic Acid Extract from *Ledum pulastre* L.. Food Sci..

[172] Sun Q., He M., Zhang M., Zeng S., Chen L., Zhou L., Xu H. (2020). Ursolic acid: A systematic review of its pharmacology, toxicity and rethink on its pharmacokinetics based on PK-PD model. Fitoterapia.

[173] Gu Z., Lin S., Yan W., Chen D., Zeng Z., Chen L., Li Y., He B. (2022). Enhanced Water Solubility and Anti-Tumor Activity of Oleanolic Acid through Chemical Structure Modification. Int. J. Mol. Sci..

[174] Gupta A., Shetty S., Mutalik S., Chandrashekar H.R., Nandakumar K., Mathew E.M., Jha A., Mishra B., Rajpurohit S., Ravi G. (2023). Treatment of *H. pylori* infection and gastric ulcer: Need for novel Pharmaceutical formulation. Heliyon.

[175] Jin Z.H., Qiu W., Liu H., Jiang X.H., Wang L. (2018). Enhancement of oral bioavailability and immune response of Ginsenoside Rh2 by co-administration with piperine. Chin. J. Nat. Med..

